# Roles of Catalysts in Plasma Conversion of N_2_ and H_2_ to NH_3_: Advances, Challenges, and Future Directions

**DOI:** 10.1021/acs.energyfuels.5c01891

**Published:** 2025-07-21

**Authors:** Parameswaram Ganji, Rok Zaplotnik, Gregor Primc, Miran Mozetič, Alenka Vesel

**Affiliations:** Department of Surface Engineering, 61790 Jozef Stefan Institute, Jamova cesta 39, 1000 Ljubljana, Slovenia

## Abstract

The increasing promotion of a hydrogen-based economy and the use of low-carbon energy sources is critical to the global drive toward carbon neutrality by 2050, and ammonia is among the most promising intermediate products. The current industrial method for ammonia synthesis is the Haber-Bosch (H–B) process, which requires large amounts of fossil fuels, high temperatures and pressures, significant capital investment, and environmental issues. An alternative research interest focusing on plasma catalysis offers a clean, sustainable, and flexible alternative method to convert nitrogen into active species for ammonia (NH_3_) synthesis, but the science of ammonia synthesis using plasma technologies is still in its infancy. In the current review, we summarize the roles of catalyst materials and different plasma-excitation methods (i.e., dielectric barrier discharge (DBD), microwave (MW), gliding arc (GA), and radio-frequency (RF)) for plasma-based ammonia synthesis. We discuss the mechanisms of NH_3_ synthesis in the presence of a plasma with a catalyst under different plasma conditions. We summarize recent developments and the key challenges related to plasma catalytic NH_3_ synthesis, scaling-up possibilities, economic concepts, and an outlook for the future. Finally, this review aims to provide a detailed overview of the emerging ammonia synthesis technologies developed to effectively store green hydrogen for future applications.

## Graphical abstract

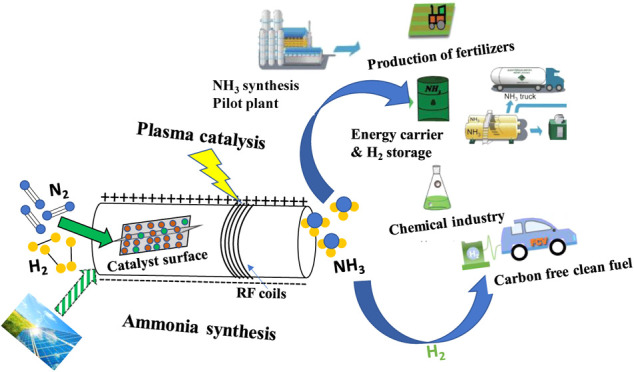

## 1 Introduction

The industrial production of ammonia (NH_3_) in a sustainable manner and carbon-free hydrogen energy carriers are currently at the center of the research community’s interest. This is a consequence of continuous population growth and, thus, a greater demand for NH_3_ production. Apart from agriculture, where ammonia-based fertilizers boost crop yields, the current focus is on the transport sector, which it is potentially a long-term, carbon-free fuel.^1,2^ NH_3_ has recently been proposed as an energy carrier because it is easier to store and transport than pure hydrogen (H_2_).^3^ The different possible applications of NH_3_, that is, for cooling, fermentation, energy storage and conversion, are shown in Figure 1a. Currently, almost 80% of industrially produced NH_3_ is converted into fertilizers such as urea, ammonium nitrates, and ammonium phosphates, which are essential for feeding the world’s growing population. However, this share of NH_3_ consumption may change significantly in the future, owing to the transition to green energy. The worldwide NH_3_ market is expected to witness significant growth in the coming years. The current global NH_3_ production is approximately 250 million tonnes per year (2024).^4^ The global NH_3_ production capacity is expected to increase from around 276 million tonnes in 2026 to almost 290 million tonnes in 2030. This projected increase in capacity is due to approximately 113 planned and announced ammonia plants worldwide, which are scheduled to come on stream by 2028. Current global NH_3_ production and forecast for the future is shown in Figure 1b.^4^ Thus, the NH_3_ market share is forecasted to be a subject of progressive growth in the near future, which will be accelerated by new paths in green energy in addition to its traditional use. However, challenges, such as the high cost of switching to greener technologies, could hamper market development.

**Figure 1:**
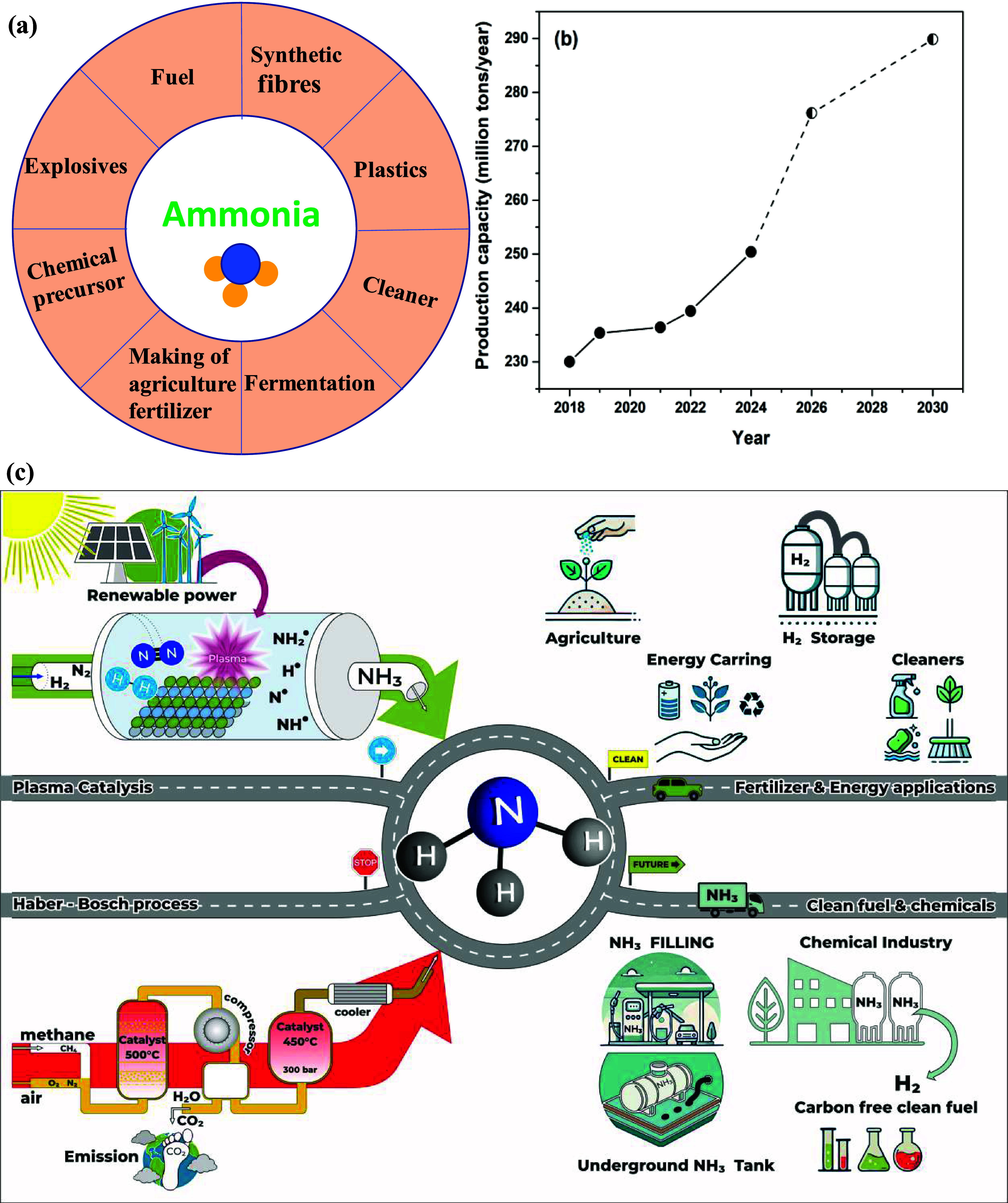
(a) Uses of NH_3_ in various real-world applications and (b) the current global NH_3_ production and forecast for the future. (c) A road-map for plasma catalysis for ammonia synthesis with a view to sustainable future applications.

The main component for NH_3_ synthesis is nitrogen (N_2_), which has some physical properties (see Table 1) that hinder the easy synthesis of NH_3_. Therefore, the only widely used synthesis method is the Haber-Bosch (H–B) process. However, the H–B process requires catalytic hydrogenation reactions at high temperatures (450–650 °C) and pressures (at least 5–20 MPa). As a result, the process is highly energy-consuming, which is why the research community is developing new alternative ways of synthesis.

**Table 1:** Nitrogen and Hydrogen Properties

	nitrogen	hydrogen
bond dissociation enthalpy	945.33 kJ mol^–1^	435.78 kJ mol^–1^
bond dissociation energy	9.765 eV	4.5 eV
electron affinity	–1.90 eV	0.754 eV
ionization potential	15.85 eV	13.6 eV
3H_2_ + N_2_ ⇌ 2NH_3_	Δ*H* = **–**45.9 kJ/mol	

Ammonia synthesis by the Haber-Bosch process was developed over a century ago, and in 1913, it was first manufactured on an industrial scale in a plant. However, this process also has several disadvantages. In addition to the requirements of high temperatures and pressures, the efficiency of this process decreases with decreasing plant size. What is even more problematic is that the H–B process is responsible for approximately 1.6% of global carbon dioxide (CO_2_) emissions. Therefore, researchers who are focusing on the social and environmental situation around the world are looking for more environmentally friendly processes to produce NH_3_.

Currently, several technologies^5,6^ have been developed to move away from the traditional H–B process: the “green” Haber-Bosch process, electrochemical, photochemical, and plasma-assisted catalysis (see Figure 2). A green H–B process has been introduced for H_2_ production from solar power to split water into hydrogen and oxygen. Researchers have also been working on H_2_ formation using the plasma-assisted photocatalytic method. Recently, more attention has been paid to plasma-catalytic ammonia production, as nonthermal plasma has received great attention for supporting NH_3_ production because of its advantages, such as mild conditions, wide feasibility, low cost, flexibility, and low energy consumption.

**Figure 2:**
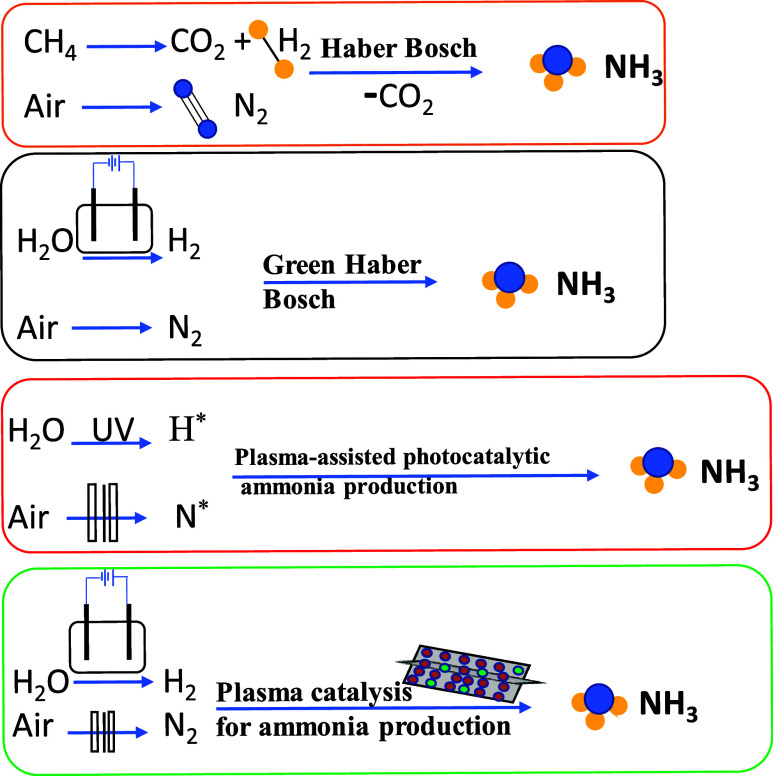
Process comparison of different NH_3_ production methods.

Currently, plasma catalysis is an emerging field for NH_3_ production, and may be a suitable replacement for the conventional H–B process. In recent decades, numerous research efforts have been made to develop nonthermal plasmas and heterogeneous catalysts of plasma species for NH_3_ synthesis. Therefore, the number of scientific publications is also increasing, indicates the growing importance of this topic, as shown in Figure 3.

**Figure 3:**
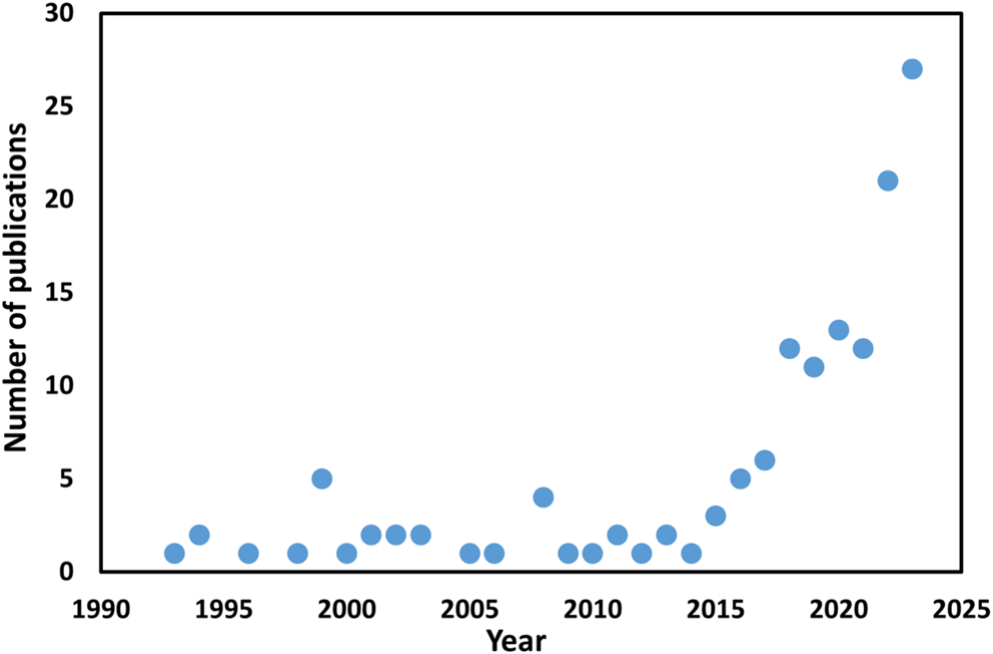
Increasing number of publications according to Web of Science. The keywords used in this search were “NH_3_ synthesis” and “plasma discharge”.

The present review addresses this critical gap by providing a comprehensive and up-to-date analysis of plasma-assisted NH_3_ synthesis, covering various plasma sources (i.e., DBD, MW, GA, and RF discharges), reactor designs, discharge conditions, and the role of plasma–catalyst interactions. Particular attention is paid to key performance indicators such as NH_3_ yield, energy efficiency (g-NH_3_/kWh) and catalyst stability, allowing direct comparison between systems. It also presents recent advances in energy optimization strategies, catalyst development and process scalability, an area that is receiving increasing attention owing to the need for practical implementation. This review article critically examines the current challenges in achieving economically viable energy efficiency and scaling up plasma catalytic systems for industrial applications. It also compares the techno-economic and energy perspectives of the H–B process and plasma-based systems, and shows where and how the new approach can be beneficial.

The unique feature of this review is that it focuses on bridging the gap between the fundamentals of plasma catalysis and applied technology perspectives, an interface where many reviews fail. By summarizing emerging trends, highlighting active catalytic materials, and identifying persistent bottlenecks, this article is intended to serve as a roadmap for researchers and engineers.

Given the increasing urgency of sustainable chemical processes and the growing global interest in green ammonia for hydrogen storage, fertilizer and fuel applications, this review is both timely and necessary. It not only summarizes the current state of knowledge in both plasma and catalyst role in the ammonia synthesis, but also sets the stage for the next generation of research by identifying future directions, research priorities and innovation opportunities. This makes it highly relevant for academic researchers, technology developers and industry representatives explore pathways to decarbonize ammonia production.

## 2 Plasma Sustained in Nitrogen, Hydrogen, and Ammonia

Gaseous plasma can be sustained in two distinctive modes, i.e., thermally equilibrium and nonequilibrium. The gas temperature governs reactions in the thermally equilibrium plasma. An appreciable degree of ionization in thermally equilibrium plasma is achieved at very high temperatures, often close to 10,000 K. Such high temperatures will ensure a high dissociation fraction of nitrogen and hydrogen molecules, but the dissociation fraction of ammonia will be also large. The dissociation fractions are governed by the gas temperature and the type of molecules introduced into the equilibrium plasma. Considering the data in Table 1, it is obvious that the dissociation fraction of hydrogen at a given temperature will be much larger than that of nitrogen because of the large differences in dissociation energies. The dissociation fraction of ammonia will be large, too. Passing a mixture of nitrogen and hydrogen through a discharge that sustains the equilibrium plasma favors the dissociation of nitrogen and hydrogen, which is beneficial. However, the ammonia molecules that are formed upon the interaction of hydrogen and nitrogen atoms are also dissociated; thus, equilibrium plasmas may not be very useful for the synthesis of ammonia. Furthermore, thermal plasmas are limited to a rather small volume; therefore, the production of any plasma-borne species is limited. Thermal plasmas are usually sustained by plasma torches powered by low-impedance discharges such as DC, RF, or MW discharges. Thermal plasmas at atmospheric or higher pressures radiate as a blackbody, and the radiation represents a loss of energy. Another loss mechanism involves cooling the gas on the surfaces of materials facing thermal plasma; thus, large gradients in the gas temperature and resultant degrees of ionization and dissociation are observed.

Nonequilibrium plasmas are usually sustained at gas temperatures between 300 and 1000 K. However, the electron temperature is much higher, often between 10,000 and 100,000 K. Electrons from the high-energy tail of the distribution function are capable of dissociating and ionizing molecules. The frequency of the dissociation and ionization collisions with these electrons depends on the electron density and electron temperature, and only marginally on the gas temperature. Therefore, the power absorbed by the gaseous plasma is used for the formation of molecular radicals in the nonequilibrium plasma. The radicals are unstable and tend to associate either in the gas phase or on the surface of the plasma reactor. The association in the gas phase should ensure the conservation of the energy and momentum of the colliding particles. Therefore, two-body collisions, such as N + N → N_2_, H + H → H_2_, N + H → NH, and NH + H → NH_2_, are impossible. Three-body collisions in the gas phase ensure conservation of energy and momentum. The reaction N + N + N_2_ → N_2_ + N_2_ and alike represent a loss of molecular radicals in nonequilibrium gaseous plasma. Excessive energy is shared between the molecules after the collision and will thus heat the gas. The probability of three-body collisions increases with the square of gas pressure. The three-body collision frequency at a pressure of 1 mbar is approximately 10 Hz (negligible) and as high as 10 MHz at atmospheric pressure. Obviously, the loss of radicals in the gas phase at low pressures is marginal.

Surface reactions are another channel for the loss of radicals. The radicals diffuse in the gas phase and eventually reach the surface of the plasma reactor, where they adsorb. The adsorbed N and H atoms will either recombine to form parent molecules or form NH_3_. The ammonia molecule is desorbed from the surface and enter the gas phase, where it is subjected to free electrons, which cause the dissociation of NH_3_ to form N, NH, NH_2_, and H radicals. The loss of synthesized ammonia molecules by electron-impact dissociation is the limiting factor that governs the overall efficiency in the production of ammonia in nonequilibrium gaseous plasma. In fact, ammonia is almost entirely degraded in nonequilibrium plasmas, and the radicals formed upon dissociation of ammonia are likely to form N_2_ and H_2_ molecules rather than associate back to NH_3_. Even moderately powerful plasmas cause almost complete degradation of NH_3_ molecules.^7^ As early as 2013, Wang et al.^8^ reported almost complete (100%) decomposition of ammonia in their plasma reactor. More recently, Van Steenweghen et al.^9^ reported 98% ammonia conversion, Andersen et al. 82%^10^ and 15%,^11^ and Gao et al.^12^ 40%. Recent achievements and some reaction mechanisms for ammonia destruction in plasma reactors have been summarized by Zhu et al.^13^

The loss of radicals depends on the gas pressure and the type of material facing the nonequilibrium gaseous plasma. At low pressure, the discharge power absorbed by the plasma is spent on heating the surface (because of the surface exothermic reactions), while at high pressure, it is spent on gas heating (by gas-phase exothermic reactions at three-body collisions). The predominant mechanism depends on the geometry of the plasma reactor and the gas pressure. As a rule of thumb, the surface loss is predominant at pressures below several 10 mbar, and the gas-phase loss is more probable at higher pressures. The surface loss is marginal at atmospheric pressure compared to the gas-phase loss. The gas-phase association of H and N atoms to form ammonia may be intensive at atmospheric pressure via three-body collisions, as is the dissociation of ammonia molecules.

The above considerations lead to the conclusion that the catalytic surface association of radicals is effective at low to moderate pressures (probably up to a few 10 mbar), while at high pressures, the gas-phase reactions are predominant, so the radicals formed in a volume between the electrodes are lost by gas-phase reactions rather than benefiting from the catalytic properties of solid materials such as catalysts. Rather uniform low-pressure plasmas will expand in an entire plasma reactor almost regardless of its size, as long as the discharge is sustained below the Paschen minimum, that is, the product of the interelectrode distance and the pressure of approximately 1 mPa. Low-pressure plasmas are characterized by a low specific power (i.e., power divided by the volume of uniform plasma). In some industrial reactors, low-pressure plasma can be sustained at a specific power as low as 1 W/liter.^14^

The plasma sustained at low pressure is always continuous, i.e., the concentration of radicals does not depend on the time or position. In contrast, atmospheric-pressure plasmas are uniform when sustained by high-frequency discharges (e.g., 1 MHz and above), whereas at lower frequencies of the electric field, they are temporally and spatially highly nonuniform. In fact, low-frequency discharge causes the formation of streamers rather than continuous plasma. The streamers will last for microseconds, and they will be limited to a small volume. They are frequently and randomly distributed in the volume between the electrodes. Consequently, the plasma appears uniform when observed with the naked eye; streamers can be recorded using a fast camera. The advantage of streamers is low power consumption because a high concentration of plasma radicals appears here and there, and the radicals diffuse in molecular gases poor with radicals, so the loss by three-body collision is suppressed. Streamers are usually sustained by a dielectric barrier discharge (DBD). The dielectric barrier limits the discharge current and prevents arcing, which would occur otherwise.

## 3 The Role of Catalysts for NH_3_ Synthesis

The chemical reaction rates on materials facing plasma can be enhanced by using catalyst materials. These catalysts can facilitate desired surface reaction. As already mentioned, there are competitive reactions on the surfaces facing plasma: (1) association of N and H atoms to form parent molecules (N_2_ and H_2_), and (2) association of N and H atoms to form ammonia. The reaction probabilities for reactions 1 and 2 depend on the type of the surface material. In one study, the conversion of N atoms to ammonia was as high as almost 50%, and the remaining N atoms were converted to N_2_ molecules.^15^ The highest production of ammonia was observed for high-purity nickel, whereas for stainless steel, it was moderate (between about 15 and 20%). Catalyst performance and activity are measured by reactant conversion, defined as the number of moles of reactant converted into products divided by the number of moles of reactant fed. The selectivity is determined by the conversion of the reactants into the desired product relative to the total conversion of the reactants.

The Sabatier principle is of fundamental importance for catalysis. Optimum activity is achieved with the correct interaction energy between the adsorbates and the catalyst. The reactants must adsorb and bind strongly enough to the catalyst surface to avoid immediate desorption but not so strongly that the resulting products cannot be desorbed. In several reactions, catalysts help reduce harmful byproducts, making the processes cleaner and more environmentally friendly.

Also, in the NH_3_ synthesis process, the catalysts play a crucial role in terms of industrial feasibility and economics. The catalysts lower the activation energy required for the reaction. This is shown in the catalyst activation energy diagram in Figure 4. In the catalyzed reactions, the energy barrier for transforming reactants to products is much lower than that without catalysts (i.e., uncatalyzed reaction). The diagram in Figure 4 is obsolete when nitrogen and hydrogen dissociate into parent atoms already in the gas phase (because the sticking probability is very large for atoms), but it may be useful when studying dissociative surface adsorption of nitrogen molecules with high potential energy, for example, N_2_ metastables or highly vibrationally excited N_2_ molecules in the ground electronic state. The potential energy of nitrogen metastables is approximately 7 eV, and the vibrational temperature of nitrogen in a nonequilibrium plasma is several 1000 K. The activity of the catalyst determines the temperature required to achieve sufficient conversion.

**Figure 4:**
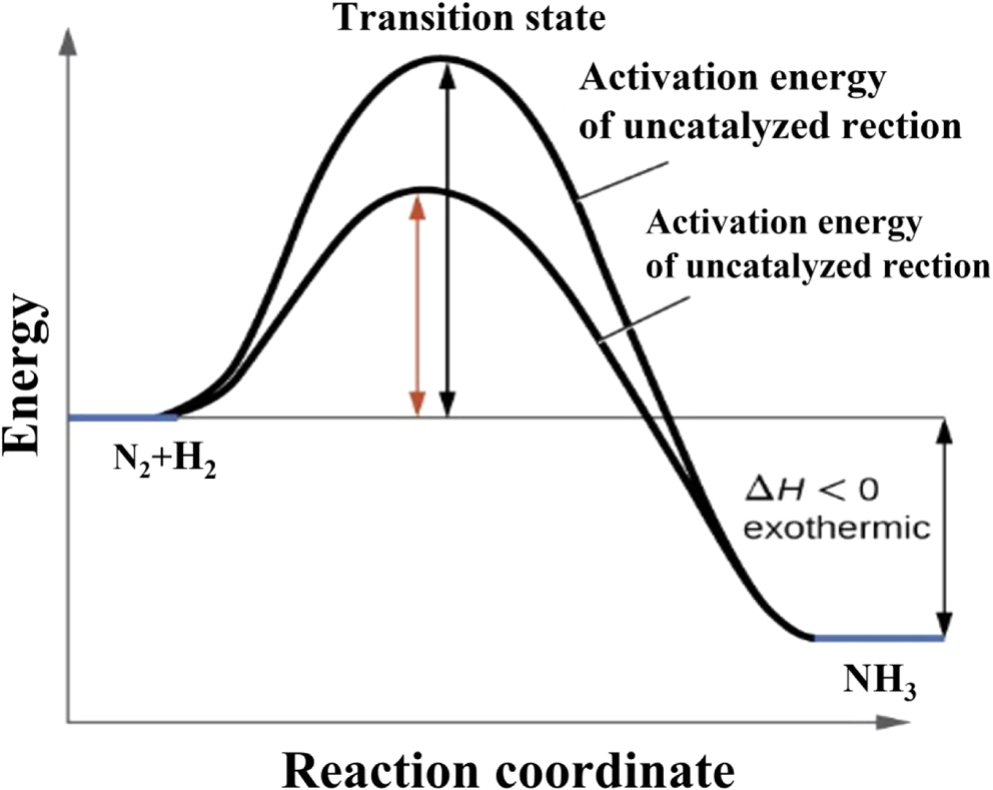
Comparison of the reaction coordinates for catalyzed and uncatalyzed NH_3_ synthesis.

For NH_3_ synthesis via the Haber-Bosch process, high temperatures (450–650 °C) and pressures (5–20 MPa) are needed. The increasing need for NH_3_ production and, consequently, the increasing number of H–B plants, where large production plants can produce approximately 2000 tons per day, led to considerable energy consumption. According to statistics, the NH_3_ synthesis industry consumes more than 2% of the global energy supply and releases over 670 million tonnes of CO_2_ annually, which is equivalent to 1.6% of total CO_2_ emissions, and to 2.5% of global greenhouse gas emissions.^16^ Therefore, to optimize energy consumption, much research has focused on the development of new catalysts and technologies that can fix nitrogen under mild conditions. The most commonly used catalyst for NH_3_ synthesis by H–B process is an iron catalyst containing a mix of Al_2_O_3_, MgO, and SiO_2_ for mechanical strength and as structural promoters, as well as some electronic promoters such as CaO and K_2_O.^17^ An ideal catalyst for this reaction should be able to stabilize the transition state for N_2_ dissociation and, at the same time, have a weak affinity for NH_*x*_ intermediates (*x* = 0, 1, or 2) so as not to hinder the further reaction and/or desorption of NH_3_. However, as these activation and adsorption energies are correlated with each other in conventional metal catalysts, it is not possible to set these energies independently of each other.

The most important advantages of catalysts for thermal NH_3_ synthesis are listed below:

- ***Lower activation energy:*** Iron or ruthenium-based catalysts, which are mostly used to reduce the activation energy of N_2_ + H_2_ to form NH_3_, lead to lower temperatures (*t*) and pressures (*p*) in the reaction process.
- ***Increased reaction rate:*** The most important step in NH_3_ synthesis is the dissociation of the N_2_ molecules at the active sites of the catalyst; this is a rate-limiting step that leads to a faster overall reaction.
- ***Higher yield:*** The catalyst increases the efficiency of the N_2_ and H_2_ conversion rate, ultimately leading to higher NH_3_ production.
- ***Energy savings:*** By using catalysts, the reaction runs at lower temperatures and pressures, which contributes to lower energy consumption and makes NH_3_ synthesis economical.
- ***Sustained operation:*** Catalysts are designed for long-term stability and enable continuous NH_3_ production in industrial environments without frequent replacement or loss of efficiency.

## 4 Plasma Catalyst for NH_3_ Synthesis

Plasma catalysis is a “clean, sustainable and flexible” alternative method for converting nitrogen into active species for ammonia synthesis. One of the most compelling advantages of plasma catalysis is its potential to completely eliminate carbon emissions from ammonia production. In addition, plasma catalysis uses nonthermal activation mechanisms that enable chemical reactions without extreme heat or pressure. This significantly reduces the risk of thermal degradation, limits the material load on the reactors and reduces the overall environmental impact. These milder operating conditions are the main advantage of plasma catalysis.

Because the important step in NH_3_ synthesis is the dissociation of N_2_ molecules, other pathways exist to achieve this. One possibility is the dissociation of nitrogen already in the gas phase. It is feasible in gaseous plasma. The combination of plasma technology with catalysts may further benefit from the collective effect being greater than the sum of their individual contributions (see Figure 5).^18^ Reactive species created in plasma may interact with catalytically active sites and enhance reaction pathways with lower activation energy, which is facilitated by the catalyst. Plasma can also improve catalyst performance by creating more reactive defect sites. More importantly, the combination of plasma and catalysts can lead to completely new reaction pathways that enable innovative and efficient chemical processes.

**Figure 5:**
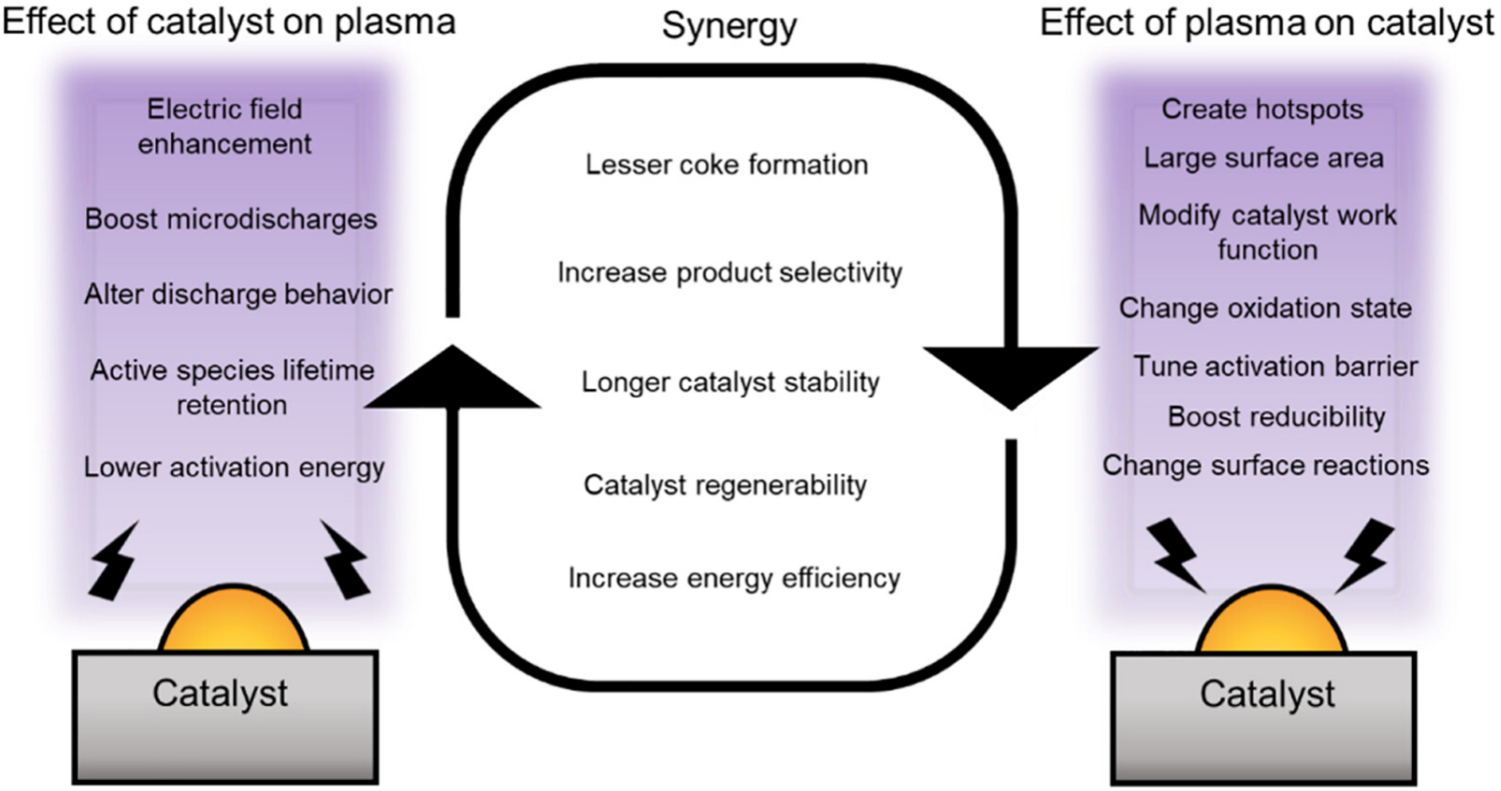
Synergy of plasma-catalytic system, Reproduced from ref 18. Copyright 2023, American Chemical Society.

Furthermore, plasma and catalyst are interrelated, as the plasma affects the properties of the catalyst, and the catalyst affects the properties of the plasma. For example, the plasma can modify the catalyst morphology or working function of the catalyst, which changes the way the catalyst works. In contrast, the presence of a catalyst in plasma may affect the discharge properties, such as the distribution of the electric field and, thus, the electron density distribution.

Mehta et al.^19^ investigated reaction equilibrium in nonthermal plasma and conventional thermal catalysis (Figure 6). His studies show that nonthermal activation reaction pathways can interrupt the equilibrium between forward and reverse reaction rates. In contrast to thermal catalysis, where forward and reverse processes reach equilibrium, nonthermal processes can drive reactions beyond this equilibrium limit. The Figure 6 reveals that, as plasma power increased, the NH_3_ yield exceeded the equilibrium threshold. However, as the temperature continued to increase, thermochemical processes regain dominance, causing the NH_3_ yield to equalize again according to the thermodynamic limits.

**Figure 6:**
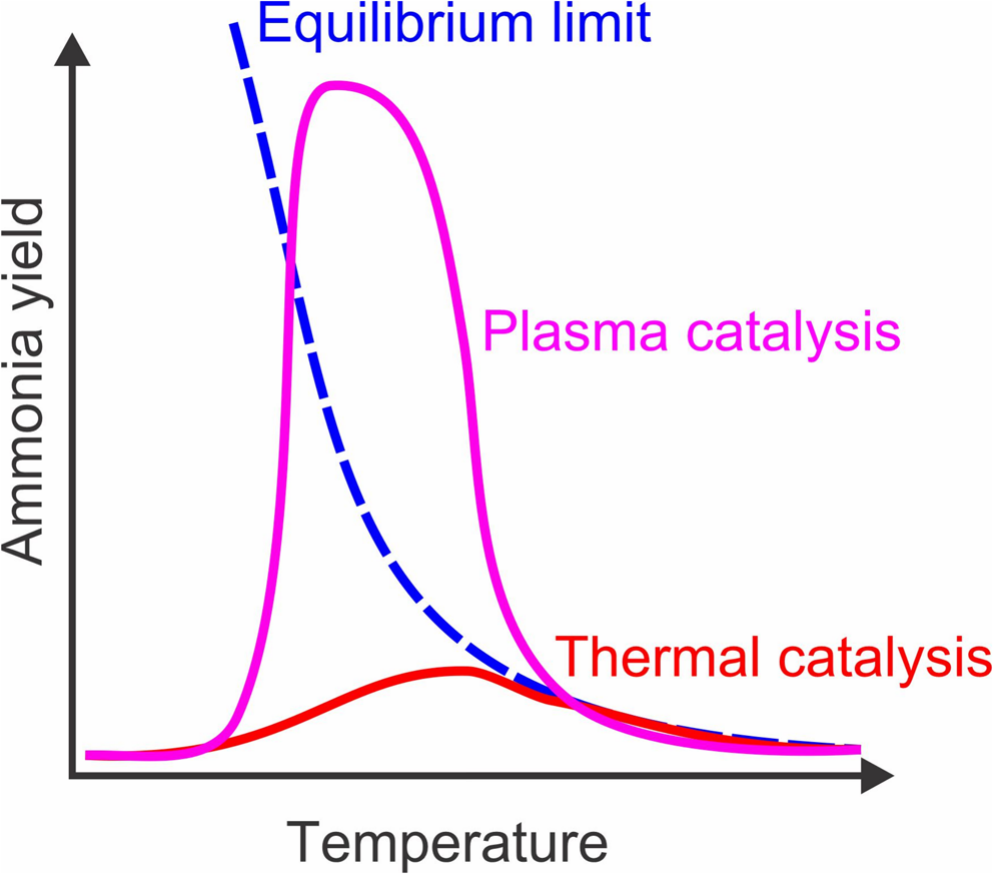
Schematic of crossing the equilibrium limited regime through the synergies between nonthermal plasma and catalysts according to Mehta, Reproduced from ref 19. Copyright 2020, American Chemical Society.

The synergy between plasma and catalysis can be evaluated in terms of the conversion, reaction rate or selectivity. In the case of NH_3_ synthesis, however, selectivity is not a significant factor, as no byproducts are formed in this reaction system except from N_2_ and H_2_ molecules. Plasma catalyst interactions and synergies remain a challenge, as the fundamental principles of plasma catalysis are not yet fully understood.^20–22^ A multidisciplinary approach is required to understand the mutual influence of plasma and catalyst.^23^ In general, the reactive plasma species can interact with the catalyst in different ways, and the catalyst may influence the conversion of nitrogen and hydrogen in the presence of plasma by utilizing the plasma radicals for reaction pathways other than the simple recombination of N and H atoms to parent molecules. Widespread research efforts have focused on the development of catalysts to enhance NH_3_ synthesis and improve NH_3_ yields. The interaction between the plasma and surface of the catalyst is considered a key factor in choosing a suitable catalyst.

## 5 Mechanisms for Plasma-Enabled Ammonia Synthesis

The reaction mechanisms of plasma ammonia synthesis are still in the development phase. Ammonia can be formed via several different reaction pathways. Several research groups have published their investigations.^5,24,25^ Zhang et al. reported that the generation of radicals by collisions of free electrons and molecules in plasma is the most important step for plasma-assisted ammonia synthesis. The specific mechanism can be described by the following reactions,^26,27^ where the dissociation of the supplied N_2_ gas is the rate-limiting step:^5^

1 $$N_{2}\to 2N$$

2 $$H_{2}\to 2H$$

3 N+H+M→NH+M

4 $$\mathrm{NH}+H+M\to {\mathrm{NH}}_{2}+M$$

5 $${\mathrm{NH}}_{2}+H+M\to {\mathrm{NH}}_{3}+M$$

Here, M represents (eqs 1–5) any molecule in the gas phase, typically N_2_ or H_2_. Equations 1 to 5 were general mechanisms that can be broken down into further detailed gas-phase^28^ and surface adsorption^29^ reactions. We give the corresponding reactions in eqs 6 to 10, where “surf” denotes the catalyst adsorption surface, “(s)” denotes the adsorption state, and “(*ν*)” denotes the vibrational excited molecule. N, H and NH_*x*_ radicals react with the catalyst surface through direct adsorption processes and adhere to the catalyst surface:^5^

6 $$N+H_{2}\left(v\right)\to H+\mathrm{NH}$$

7 $$N+\mathrm{surf}\to N_{\left(s\right)}$$

8 $$H+\mathrm{surf}\to H_{\left(s\right)}$$

9 $$\mathrm{NH}+H_{\left(s\right)}\to {\mathrm{NH}}_{2\left(s\right)}$$

10 $${\mathrm{NH}}_{2\left(s\right)}+H_{\left(s\right)}\to {\mathrm{NH}}_{3}$$

In contrast, several chemical pathways in plasma synthesis. Two main mechanisms are illustrated in (Figure 7). In the Eley–Rideal (E–R) mechanism, only one of the reactants (H or N) is adsorbed on the surface, whereas the other reactant interacts with the adsorbed species directly from the gas phase, followed by desorption of the reaction product. In the second mechanism, called Langmuir–Hinshelwood (L–H), both reactants adsorb to the surface before a reaction occurs, leading to the formation of NH_3_.

**Figure 7:**
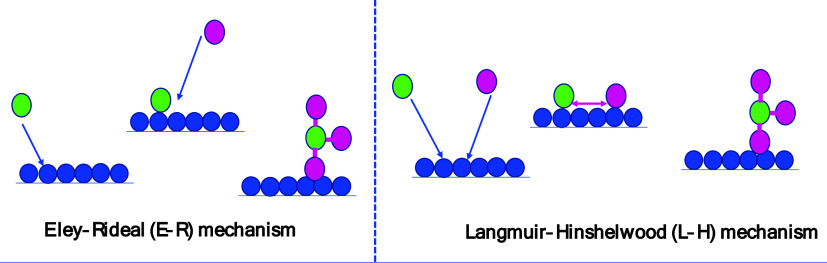
Schematics of ammonia formation surface reactions via E–R interaction (left) and L–H interaction (right).

Qu et al. mentioned some other reactions.^30^ Another interesting observation was reported by Wang et al.^24^ The authors published a possible reaction mechanism for ammonia synthesis via plasma catalysis using Ni/Al_2_O_3_ catalyst. The authors considered the well-known fact that the plasma produces many different reactive species, including atoms (N and H), ions (predominantly N_2_^+^, H_2_^+^), vibrationally and electronically excited molecules (N_2_* and H_2_*), which react with each other either in the gas phase or on the catalyst surfaces. Some electronically excited states on neutral nitrogen molecules are metastable, therefore, their radiative lifetime are orders of magnitude longer than the time between two collisions, and quenching at collisions with other plasma particles is likely to occur. It is assumed that the catalysts placed in the plasma enhance the surface reactions by generating more NH_*x*_ on the catalyst.

Rouwenhorst et al.^25^ found that the process of NH_3_ synthesis is promoted by vibrationally excited reactant molecules and termed it “plasma-enhanced catalytic synthesis”. This reaction pathway is an example of how plasma and catalysts synergistically interact for the optimal production of NH_3_. The authors considered the well-known fact that N_2_ is vibrationally excited in plasmas (i.e., N_2_(*v*)), and dissociation of N_2_(*v*) occurs at the active sites of the catalyst surface (i.e., breaking of the chemical bond in nitrogen molecule). Mehta et al. proposed a similar approach.^31^ Molecule H_2_ dissociates at the surface driven by the catalytically active surfaces. Subsequently, the adsorbed atoms combine to yield of NH_3_ following the Langmuir–Hinshelwood model.

The authors also reported two pathways: (i) surface-enhanced plasma-assisted synthesis, i.e., the N^•^ and H^•^ radicals generated in the plasma are first adsorbed on a surface (i.e., an active metal surface), where the adsorbed species (i.e., N and H atoms) are stabilized by electrons from the surface^32^ and (ii) plasma-enhanced semicatalytic pathway, i.e., N^•^ radicals produced in the plasma are first adsorbed on a surface and later recombine with H atoms produced by dissociation of H_2_ molecule on the catalytically active surface. The adsorbed N and H atoms then form NH_3_. Consequently, H* or NH_x_* poisoning (i.e., absorbed H-containing species occupying and blocking the catalytically active metal surfaces) is expected on the catalyst surface. This is a result of the imbalance of reaction rates (i.e., uncatalyzed N_2_ dissociation versus catalyzed H_2_ dissociation), hence the term “semi-catalytic″.

All these mechanisms show that the catalyst plays an important role in plasma-assisted ammonia synthesis, but this needs to be further investigated to prove the exact effect of the catalyst. Researchers are still working on mechanistic and kinetic modeling studies to fully understand it. The research community has investigated the catalytic effect of plasma catalysis in ammonia synthesis.

## 6 Different Discharges Suitable for Plasma-Catalytic Conversion

This section provides an overview of the most significant developments in plasma-driven NH_3_ synthesis, with attention to different plasma configurations and catalysis, to show how recent understanding can help in the further progress of the field. Current publications are mostly focused on NH_3_ synthesis, where the following discharges are used to sustain gaseous plasma in a mixture of nitrogen and hydrogen: dielectric barrier discharge (DBD), microwave (MW), gliding arc (GA), and radio-frequency (RF). The NH_3_ conversion and energy yields are compared, and the state-of-the-art is described.

### 6.1 Dielectric Barrier Discharges (DBDs)

A dielectric barrier discharge (DBD) consists of two opposite electrodes, at least one of which is covered with a dielectric barrier material (often quartz or alumina). An alternating voltage (AC) is applied to the electrodes. The voltage amplitudes are usually on the order of a few kilovolts, and the frequency is typically in the kHz range. The system operates at atmospheric pressure. The dielectric limits the amount of charge transported between the electrodes and, thus, the electric current, preventing the discharge from undergoing a transition into a thermal regime (sparks or arcs). The DBD always produces streamers, i.e., numerous temporally and spatially limited discharges. DBD plasma offers several key advantages, including the ability to operate at atmospheric pressure, a relatively low gas temperature, and the ability to modify a wide range of materials.^6^

The electrodes are usually two parallel plates (Figure 8), but two concentric cylindrical electrodes are most often used for gas conversion applications.^3^ Such a reactor usually consists of an inner electrode surrounded by a dielectric tube, resulting in a gap of a few millimeters, and an outer electrode, which is typically a mesh or foil wrapped around the dielectric tube. One of the electrodes is connected to a power supply, while the other electrode is usually grounded. The gas flows from one side, is gradually converted along its way through the gap between the inner electrode and the dielectric tube, and flows out the other side.

**Figure 8:**
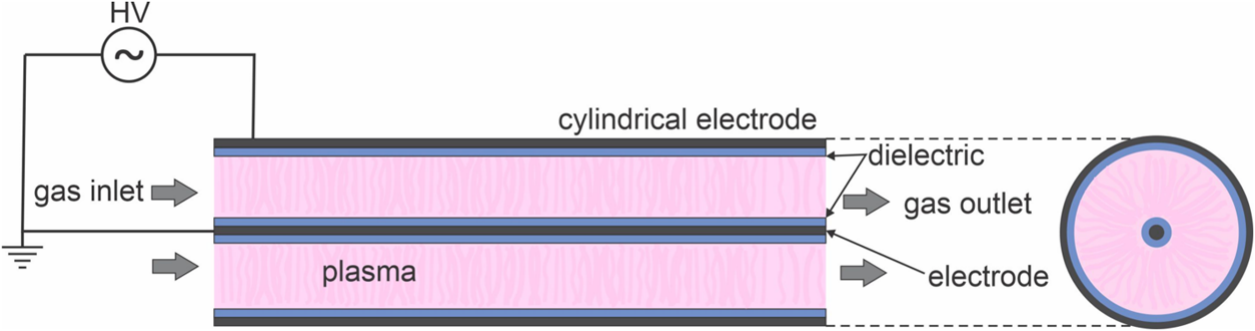
General parallel-plate DBD reactor. The dielectric barrier could be only on one electrode.

Plasma-driven NH_3_ synthesis was first independently reported by Morren and Perrot in a DBD reactor in 1859 ^33^ two years after the first report of plasma-driven conversions with a DBD reactor by Werner von Siemens.^34^ Research into plasma-assisted catalytic NH_3_ synthesis began in 1980, when the research group of Botchway and Venugopalan first investigated the effect of an iron catalyst immersed into DBD plasma for NH_3_ production.^35^ Later, plasma-assisted NH_3_ synthesis has been explored for various catalytic materials such as metal oxides,^36^ transition metals,^37^ porous catalysts,^38^ bimetallic materials,^39^ and perovskites.^40^ In addition to experimental work, studies on the reaction mechanism of plasma-catalytic NH_3_ synthesis on stainless-steel walls were conducted using X-ray photoelectron spectroscopy (XPS). Therefore, the surface of the stainless-steel wall acts as a catalyst in ammonia formation.^41^

Table 2 presents an overview of the current state-of-the-art regarding the different catalyst materials used in DBD discharges. The plasma discharge conditions and energy yields are also presented. The performance of DBD systems for NH_3_ synthesis depends on the catalyst materials. Table 2 clearly shows that the efficiency of the catalyst materials in terms of the energy yield and NH_3_ synthesis rate is a crucial indicator. The influence of the discharge power reported in Table 2 on the energy and NH_3_ yields is shown in Figure 9. The points are scattered in Figure 9a; therefore, no correlation between the energy yields and reported discharge power was observed. The large scattering of the results indicates that different reactors enable various energy yields. One would expect increasing in NH_3_ yield with increasing discharge power. Figure 9b partially supports this expectation, but the number of measured points is too low to enable a decisive conclusion. According to the above discussion, the yield should depend on the fluxes of plasma species onto the catalyst surface and the post-dissociation of ammonia molecules; however, the fluxes were not reported, and the dissociation of released NH_3_ by plasma electrons was not mentioned in the reviewed reports.

**Figure 9:**
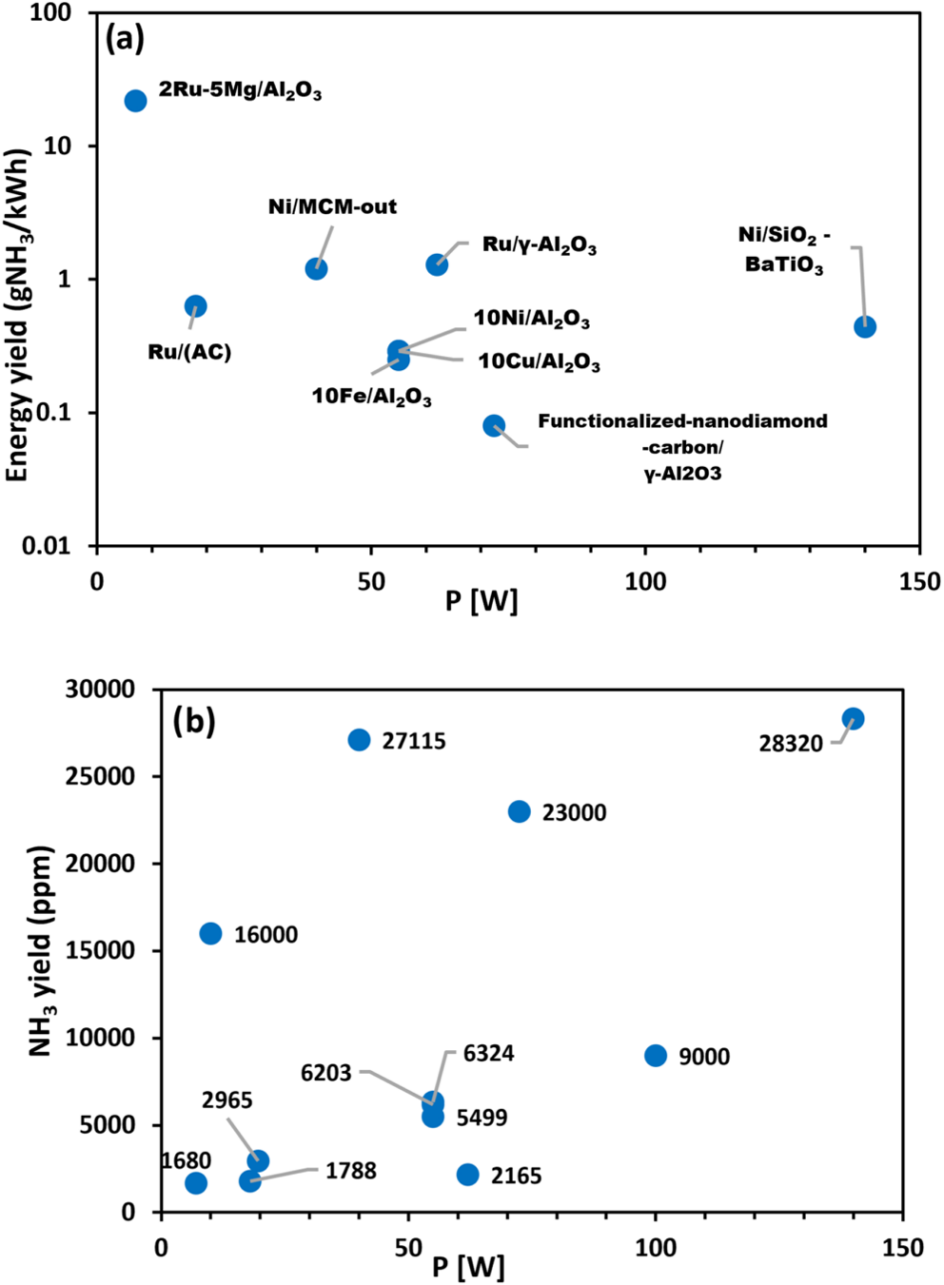
Energy yield (a) and NH_3_ yield (b) versus discharge power for DBD systems.

**Table 2:** Comparison Table for NH_3_ Synthesis by DBD Plasma

s. no	catalyst/(catalyst amount)	catalyst synthesis route	plasma reactor type	H_2_:N_2_	frequency (kHz)	power (W)	NH_3_ yield (ppm)	energy yield (gNH_3_/kWh)	refs
1	Ru/Ti-CeO_2_ (6.19 gcm^–3^)	impregnation	coaxial DBD reactor	1:2	20	19.6	2965 (174.4 μmol/g)		^44^
2	Ru/γ-Al_2_O_3_ (0.5 g)	incipient wetness impregnation	packed-bed DBD reactor	1:2	9.2	62	2165 (127.3 μmol/g)	1.29	^45^
3	2Ru-5Mg/Al_2_O_3_ (17.1 g)	impregnation	DBD reactor	1:4		7	1680 (98.8 μmol/g)	21.86	^46^
4	Ru/(AC) activated carbon (0.2 g)	ultrasonic-enhanced wet impregnation method	DBD reactor	1:1	10.1	18	1788 (105.1 μmol/g)	0.63	^47^
5	Ru+Cs/MgO (NA)	impregnation	tubular nonthermal plasma reactor system	1:3	8	10,000 Hz and 6000 V	24,100 ppm^b^ (1417.6 μmol/g)	2.3	^27^
6	γ-Al_2_O_3_		DBD reactor at atmospheric pressure	3:1	21	36 V_pk–pk_, 21 kHz	2660 (156.4 μmol/g)		^51^
7	functionalized-nanodiamond -carbon/γ-Al_2_O_3_		DBD reactor (atmospheric pressure)	3:1	1	72.4	23,000 ppm^b^ (1352.9 μmol/g)	0.08	^52^
8	CoLa/Al_2_O_3_ (16 g)	wet impregnation	DBD reactor	1:1	23.5	100	9000 (529.4 μmol/g)		^53^
9	Ni/MCM-out (0.5 g)	controlled deposition	coaxial DBD reactor	3:1	9.2	40	27,115 (1595 μmol/g)	1.2	^54^
10	10Ni/Al_2_O_3_ (2 g)	incipient wetness impregnation	coaxial DBD reactor	2:1	9.2	55	6324 (372 μmol/g)	0.29	^24^
11	Ni/SiO_2_-BaTiO_3_ (20 g)		DBD reactor	3:1	20	140	28,320 (1665.8 μmol/g)	0.44	^56^
12	10Cu/Al_2_O_3_ (2g)	incipient wetness impregnation	coaxial DBD reactor	2:1	9.2	55	6203 (364.8 μmol/g)	0.29	^24^
13	10Fe/Al_2_O_3_ (2g)	incipient wetness impregnation	coaxial DBD reactor	2:1	9.2	55	5499 (323.4 μmol/g)	0.25	^24^
14	Pt/Al_2_O_3_ (NA)	commercial	DBD reactor	1:2		10	16,000^a^ (941.1 μmol/g)		^19^

a Recalculated.

As shown in Table 2, various metal catalysts have been investigated for NH_3_ synthesis. A few studies have focused on ruthenium-based catalysts (Ru), which contribute to a relatively higher energy yield. Ruthenium-based catalysts have been selected as promising catalysts because of their excellent performance in conventional, i.e., thermal NH_3_ synthesis, which has been demonstrated by several research groups.^42,43^ Recently published Ru/Ti-CeO_2_ ^44^ shows the highest NH_3_ synthesis among all these published catalysts (Ru/γ-Al_2_O_3_,^45^ 2Ru-5Mg/Al_2_O_3_,^46^ Ru/activated carbon,^47^ Ru–Mg/γ-Al_2_O_3_ ^48^) when using coaxial DBD reactor with H_2:_N_2_ = 1:2. All catalysts were prepared by a simple impregnation method and incipient wetness impregnation, i.e., techniques often used for catalyst material synthesis, which shows that these catalysts are very easy to commercialize as long as the high cost of Ru is not an obstacle.

When the catalytic metals (especially Ru) were deposited on the alumina, the hydrogenation of the adsorbed nitrogen was accelerated to increase the NH_3_ yield.^45,49^ Peng et al.^27^ reported a novel and environmentally friendly approach for catalytic NH_3_ synthesis using Ru catalyst with carbon nanotube support and Cs promoter. They used a plasma reactor with an applied voltage of 6000 V and a frequency of 10,000 Hz. Using the N_2_:H_2_ ratio of 3:1 they produced the maximum NH_3_ yield of 24,100 ppm and obtained an efficiency of 2.3 gNH_3_/kWh. They found that Cs readily donate electrons to Ru, and Ru transfers the extra electron to the adsorbate, i.e., the N_2_ molecules adsorbed on the surface in the NH_3_ production process. The authors suggested that the electron can enter the N_2_ antibonding state via the d-orbital of Ru, which weakens the triple bond and ultimately leads to dissociation. The authors also commented on literature reports^36,50^ that MgO particles in combination with metal-based catalysts (Ru) lead to strong surface discharges, which are expected to favor the dissociation of N_2_ and H_2_ and promote the production of NH_3_. According to the author, the discharge electrodes were set on the surface of a dielectric plate, and then a MgO catalyst was smeared on their surfaces.

Mizushima et al.^49^ reported the role of Ru in the catalyst in the hydrogenation of adsorbed nitrogen through “spillover” reactions to deliver a hydrogen atom to an adsorbed nitrogen atom, as shown in Figure 10. In this study, a DBD plasma reactor was used. A metal-loaded membrane-like alumina tube was placed between the electrodes, which served as a catalyst for plasma synthesis of NH_3_ at atmospheric pressure. The authors first investigated how the introduction of pure alumina into the N_2_–H_2_ plasma led to an increase in NH_3_ yield. Finally, they tested different metal-loaded (Ru, Pt, Ni, and Fe) membrane-like catalysts for NH_3_ synthesis and concluded that the Ru-based catalyst was the most active. A schematic of the author’s illustration is shown in Figure 10. Atomic N (adsorbed) species are produced on alumina through the dissociative adsorption of N_2_* (metastable) molecules excited by electron impacts. Activated hydrogen species such as H atoms and H_2_* molecules, are produced during discharge. These hydrogen species react with N­(adsorbed) atoms directly or via adsorption on alumina to form NH_3_. When Ru is present on alumina, NH_3_ is also produced by the reactions of N­(adsorbed) atoms with H­(adsorbed) atoms on Ru. This reaction is relatively faster, and thereby, a larger amount of NH_3_ is obtained.

**Figure 10:**
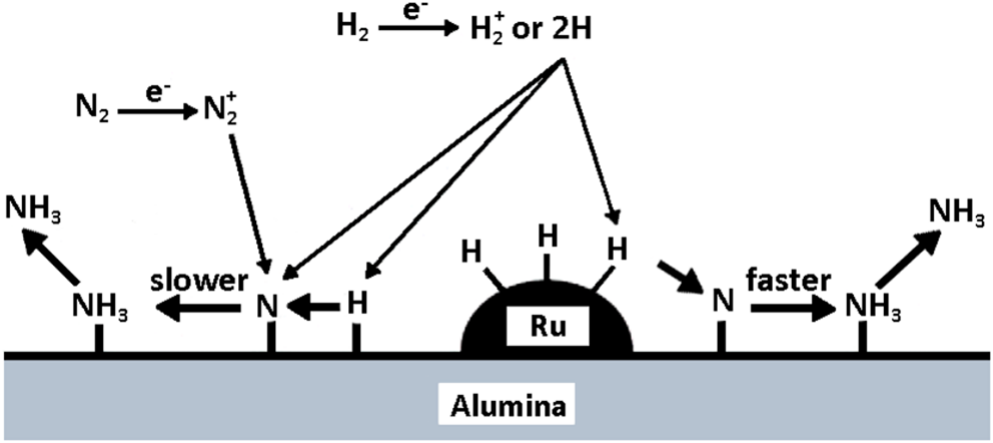
Ammonia synthesis mechanism with the assisted hydrogenation by Ru, Reproduced or adapted with permission from ref 49. Copyright 2006, Springer Nature.

Kim et al.^46^ studied a combination of pelletized 2Ru-5Mg/Al_2_O_3_ catalyst (diameter, 3.2 mm; length, 3.6 mm; surface area, 98.6 m^2^/g) and pulsed DBD plasma operated at a temperature of 300 °C for the synthesis of NH_3_ and achieved an ammonia yield of 1680 ppm and an energy efficiency of 35.7 g-NH_3_/kWh. The catalysts used in previous studies (Table 2) were generally powders and wires. Prior to 2014, studies on the nonthermal plasma synthesis of NH_3_ mainly used powder and wire catalysts. After 2014, pellet-type catalysts were introduced and used by most researchers. Kim et al.^46^ mentioned that powder catalysts are not suitable for the plasma process because they tend to spread all over the reactor tube after plasma ignition.

Zhu et al.^47^ reported Ru-based catalysts with different supports (activated carbon (AC), α-Al_2_O_3_, ZSM-5 and SiO_2_) for the synthesis of NH_3_ in a DBD plasma reactor. The presence of Ru-based catalysts boosted the NH_3_ yield and energy efficiency by 163–387% compared with using plasma alone with a specific energy input (SIE) of 5.4 kJ/L. The authors found the following sequence of catalysts for synthesis performance: Ru/AC > Ru/ZSM-5 > Ru/α-Al_2_O_3_ > Ru/SiO_2_. Thus, the type of catalyst support directly affects the performance of plasma-catalytic NH_3_ production. Due to the physicochemical properties, i.e., the higher specific surface area (1333.8 m^2^ g^–1^) of the AC support, this value provides more adsorption sites for the reactants and intermediates, including N and H radicals, excited N_2_ species, etc. Thus, the residence time of these species on the surface of Ru/AC is prolonged compared to other supported catalysts (α-Al_2_O_3_, ZSM-5, and SiO_2_), leading to a higher probability of effective collisions for NH_3_ formation. Zhu et al.^47^ also confirmed that the optimal feed molar ratio of N_2_ and H_2_ is 1:1. Low flow rates of gases were important to get higher NH_3_ yields. The authors made a final conclusion that the Ru/AC catalyst has very good performance because of the larger specific surface area, pore volume and stronger basicity of the catalyst Ru/AC.

Hessel et al.^51^ investigated γ-Al_2_O_3_ and observed a better NH_3_ yield when the N_2_/H_2_ feed ratio was ⩾2 instead of the stoichiometric feed ratio of 0.33. They produced 3500 ppm of NH_3_ at a flow of 0.4 l min^–1^, with an energy efficiency of 1.23 gNH_3_/kWh. The authors found that γ-Al_2_O_3_ produced the highest amount of NH_3_ compared with an empty plasma reactor, yielding an additional 95% NH_3_. They also commented that the addition of 2–5 vol % of Ar to N_2_/H_2_ increased the final NH_3_ concentration and energy efficiency by 2%. Another observation concerns the addition of transition metals to γ-Al_2_O_3_ to achieve higher NH_3_ yields and energy efficiency.

The functionalized nano diamond-carbon/γ-Al_2_O_3_ catalyst also actively participated in NH_3_ synthesis. The authors stated that the results indicated that carbon-based coatings can have a significant impact on plasma catalysis. However, carbon is etched in N_2_–H_2_ plasmas and forms hydrogen cyanide, which might be an obstacle. These effects may be due to changes in plasma parameters as well as changes in surface reactions. These data indicate that γ-Al_2_O_3_ is a very good support material for catalysts and higher NH_3_ synthesis.^52^

Ndayirinde et al.^53^ investigated the catalytic system CoLa/Al_2_O_3_. A more than 4-fold increase in NH_3_ concentration was observed compared to that in an empty reactor. In both cases, the H_2_:N_2_ ratio was 1:1 and the flow rate were 100 sccm. The authors confirmed that the performance of plasma-catalytic NH_3_ synthesis can be tuned to the desired mode using catalysts that change the plasma properties. The design of the plasma reactor for NH_3_ synthesis by DBD, used by Ndayirinde et al.^53^ is shown in Figure 11. It consists of an inner ground electrode (stainless steel, 8.0 mm diameter) arranged in the center of a hollow ceramic cylinder marked as “dielectric barrier” in Figure 11. Notably, the plasma properties (power and current profile) varied significantly depending on the catalyst marked as “packing” in Figure 11. These results show that the addition of the catalysts alters the discharge properties and thus plasma chemistry, probably because the catalyst is packed in the volume between the electrodes.

**Figure 11:**
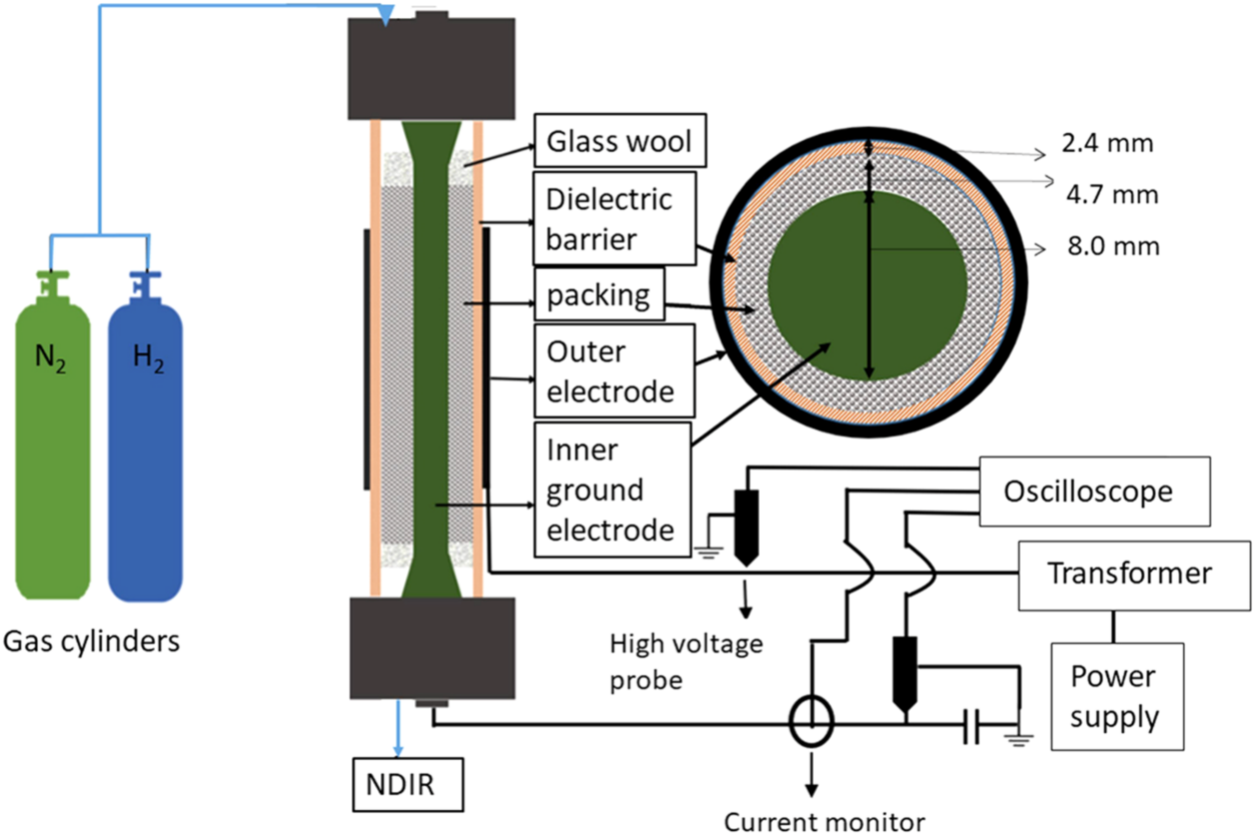
Schematic representation of the DBD reactor used for plasma-catalytic NH_3_ synthesis, Reproduced or adapted with permission from ref 53. Copyright 2023, Elsevier Inc.

Compared with the Ru catalyst, a higher NH_3_ concentration with a lower energy yield (<1 gNH_3_/kWh) could be achieved with Ni and other metals. Because noble-metal catalysts are expensive, a catalytic system with nickel supported on Mobil Composition of Matter (MCM), i.e., Ni/MCM-out, shows promising performance. At a low flow rate of 40 sccm, a high NH_3_ yield of 5.3%, NH_3_ concentration of 27,115 ppm, and energy yield of 1.2 gNH_3_/kWh was obtained. The authors carried out very long reaction times (up to 150 h) to investigate the stability of the Ni/MCM-out catalyst (Figure 12). The catalyst was found to be very stable under plasma conditions, with the final and initial rates of NH_3_ (R_NH3_) values (i.e., 5380 ± 121 μmol/g/h in the last 150 h vs 5630 ± 118 μmol/g/h in the first 20 h) being quite comparable (Figure 12).^54^

**Figure 12:**
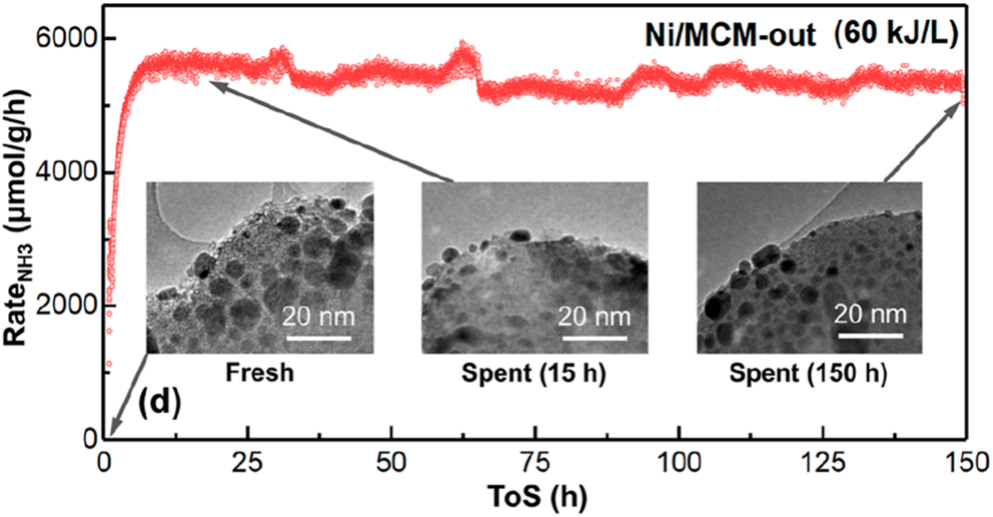
R_NH3_ rate for Ni/MCM-out catalyst as a function of reaction time (ToS) for 150 h, and HRTEM images of fresh and used catalysts, Reproduced from ref 54. Available under a CC-BY 4.0 license. Copyright 2022, Yaolin Wang.

Wang et al.^24^ reported that the highest NH_3_ synthesis rate of 6324 ppm was achieved with Ni/Al_2_O_3_, which was 100% higher than that of plasma only. The authors commented that the presence of Ni/Al_2_O_3_ could produce more radicals (e.g., N, H, and NH) in the gas phase by enhancing the reduced electric field, and also promote surface reactions by generating more NH_*x*_ through stepwise hydrogenation via the Langmuir–Hinshelwood (L–H) mechanism.

In another type of Ni-supported catalyst system, the Ni catalyst was coated onto carrier materials such as SiO_2_/tetraethylenepentamine (TEPA) to increase the surface area and improve the reaction rate.^55^ Akay et al.^56^ found that highly reactive species of hydrogen and nitrogen generated by the combination of dielectric-barrier material and porous catalyst in a DBD enable NH_3_ synthesis at relatively low temperatures and atmospheric pressure. In addition, the use of higher dielectric constant materials, such as BaTiO_3_ spheres in the discharge gap, can enhance the NH_3_ concentration and nitrogen conversion with reduced energy consumption. Finally, the authors stated that, by using this catalyst, the energy cost could be decreased by approximately 20%, whereby the energy costs increased with increasing catalyst mass (over 5 wt %). Figure 13 shows SEM images of the Ni/SiO_2_ catalysts. A highly porous structure was observed, which enabled diffusion path lengths that were comparable to the pore size.

**Figure 13:**
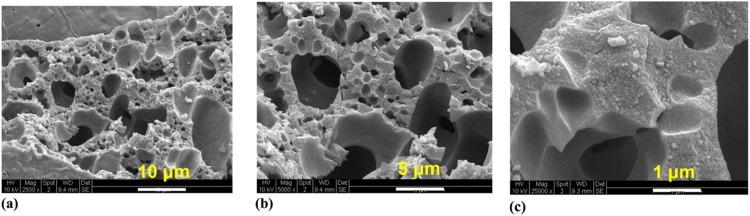
SEM images of the SiO_2_ supported catalyst Reproduced from ref 56. Copyright 2017, American Chemical Society.

In addition to Ni catalysts, other studies have focused on Cu- and Fe-metal catalysts. Wang et al.^24^ investigated industrially applicable Cu and Fe catalysts supported on Al_2_O_3_, of which Cu/Al_2_O_3_ gave a higher yield of 6203 ppm. Finally, the authors found that the order of the various transition-metal catalysts supported on Al_2_O_3_ further enhanced the NH_3_ synthesis rate. The catalyst activity decreased in the following order: Ni/Al_2_O_3_ > Cu/Al_2_O_3_ > Fe/Al_2_O_3_ > Al_2_O_3_ > plasma only.

Mehta et al.^19^ reported different metal-contained plasma catalysts for NH_3_ synthesis in a DBD reactor packed with Al_2_O_3_, Ni/γ-Al_2_O_3_, and Pt/γ-Al_2_O_3_. The following conditions were used: total gas flow rate of 40 mL min^–1^ and N_2_: H_2_ ratio of 2:1. The main reason for selecting these materials is that they bind N more weakly than the optimal thermal catalyst Ru (the N-metal bonding strength follows the order Ru > Ni > Pt). The authors conducted a kinetic analysis and found that these materials exhibited behavior beyond equilibrium. This indicates that the reaction kinetics are not completely governed by conventional equilibrium principles, suggesting improved or altered performance characteristics under specific conditions. According to the authors, the temperature sweep profiles of the Pt catalyst were similar to those of the background experiments. The background measurements were performed in a reactor packed with bare support material (100 mg of γ-Al_2_O_3_), as opposed to an empty reactor, because dielectric packing can influence the discharge characteristics (plasma volume, discharge gap, etc.). According to the author, however, the empty reactor behaves similarly to a γ-Al_2_O_3_-packed reactor. The NH_3_ yield continued to increase with increasing temperature (Figure 6) and significantly exceeded the bulk equilibrium limit before eventually decreasing. Pt increased NH_3_ production compared to the background at temperatures up to approximately 1000 K, after which the yields converged.

### 6.2 Microwave (MW) Plasmas

Microwave plasma is created by applying microwaves with a frequency between 300 MHz and 10 GHz to a gas without the use of electrodes. Such a high frequency enables the plasma to be in a continuous mode, i.e., without streamers. There are different types of MW plasmas:^57^ freely expanding atmospheric-pressure plasma torches, electron cyclotron resonance plasmas, and plasma sustained by surface wave discharges. The latter is most frequently used in gas-conversion applications.^3^ The gas flows through a quartz tube that is transparent to MW and intersects with a rectangular waveguide to sustain the discharge (Figure 14). Microwaves propagate along the interface between the quartz tube and plasma column, and microwave energy is absorbed in the sheath between the glass tube and plasma. It should be stressed that the penetration of microwaves in a conductive medium, such as plasma, is limited by the skin effect. MW plasmas can be operated at reduced pressure (from approximately 10 Pa, provided that the sheath thickness is smaller than the plasma thickness) up to atmospheric pressure and beyond. When operating at low pressure, they exhibit very good energy efficiency, but when operating above 0.1 bar, they approach thermal equilibrium with gas temperatures of a few 1000 K, which greatly reduces their energy efficiency. In the latter case, the discharge energy is spent on heating the gas at high pressures, as explained in Section 2.

**Figure 14:**
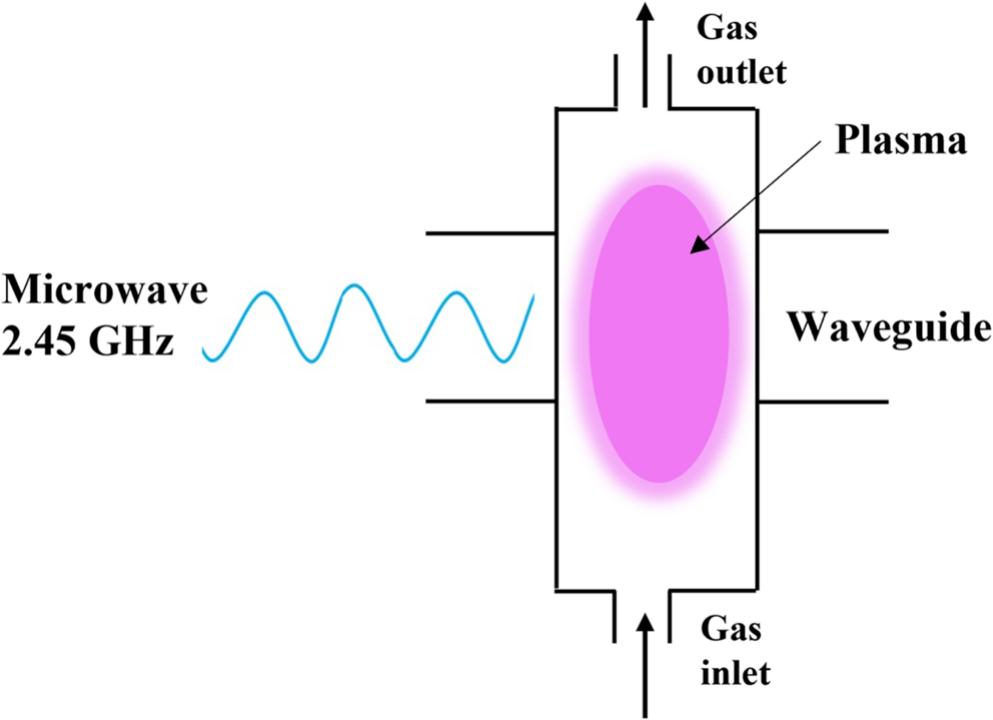
Schematic illustration of the MW plasma reactors most often used for gas conversion applications.

Nakajima et al.^58^ studied NH_3_ formation in MW discharge at atmospheric pressure and two types of hydrogen injections (i.e., into the afterglow region or directly into glowing plasma). The MW generator was operated at a maximum power of 1.3 kW and a frequency of 2.45 GHz. The authors found that gas injection was the most significant factor. According to Nakajima et al.^58^ the quenching of nitrogen metastables in the afterglow region led to an enhancement of the NH_3_ formation.

Bai et al.^59^ synthesized NH_3_ from plasma created in CH_4_/N_2_ gas mixture using catalysts Co/γ-Al_2_O_3_ and Co–Fe/γ-Al_2_O_3_. During NH_3_ production, valuable byproducts such as carbon nanotubes (CNTs) were formed, increasing the overall economics of the process. When calculating the total NH_3_ production during the 30 min reaction period, it was found that the NH_3_ production in MW plasma was 143.5% (383.1 × 10^–8^ mol) higher with the catalyst than that with plasma only (157.3 × 10^–8^ mol). The authors commented that by utilizing MW plasma, novel reaction pathways could be enabled, offering scalability and economic feasibility for future industrial applications. The authors did not report the formation of hydrogen cyanide, which should be intensive, taking into account the very strong triple C–N bond, so the HCN is not as likely to be fully dissociated, such as NH_3_.

Tanaka and his research group^37^ published the effect of catalysts in microwave discharge plasma at the pressure of 650 Pa for ammonia synthesis using iron and molybdenum wires as catalysts. They found that microwave (MW) discharge plasma showed a very good NH_3_ production rate compared to a reactor without a catalyst. The authors explained that the generation of plasma radicals depends on the discharge type. The RF discharge caused the adsorption of H and NH_*x*_ radicals to form ammonia and hydrazine, and the MW discharge caused the formation of ammonia by the reaction between adsorbed N atoms and/or metal-N and H, H_2_, and NH from the plasma.

Kiyooka et al.^41^ reported the synthesis of NH_3_ in the Electron Cyclotron Resonance (ECR) plasma, where the reaction schemes were clarified using surface analysis of the catalyst and plasma diagnostics. A MW power of 200 W, and gas composition of N_2_ (75 vol %) and H_2_ (25 vol %), an operational pressure of 6 × 10^2^ Pa and an exposure time of 2 h were specified as plasma processing conditions. The dissociative adsorption of N_2_ molecules and/or molecular ions as well as the adsorption of NH_*x*_ occurred on the stainless-steel wall of the reaction chamber (according to the author). This process is an essential part of plasma-catalyzed reactions, in which surface interactions help to break strong bonds (Fe–N bonds) and promote further reactions leading to NH_3_ synthesis. In addition, the adsorption of NH_*x*_ radicals was absent when the stainless-steel wall surface was covered with aluminum foil. Thus, the surface of the stainless-steel wall acted as a catalyst for ammonia formation. These adsorption processes play an important role in determining the overall productivity and mechanism of the NH_3_ reaction system.

A recent publication on the use of cyclic MW plasma for NH_3_ synthesis using a novel reactor was developed by Brown et al.^60^ The authors used MW plasma to pretreat Fe, Mn and CoMo particles for chemical looping ammonia synthesis (CLAS) under typical thermocatalytic conditions. In contrast to the DBD plasma, the catalyst bed in the MW plasma is located outside the plasma generation zone; therefore, external heat input is possible. The authors concluded that MW plasma allows for higher NH_3_ production rates due to the generation of vibrationally activated nitrogen, which improves the reactivity of the catalyst surface. Simple, economical, and environmentally benign Fe-based metallic catalysts were used for the synthesis of NH_3_ under atmospheric pressure. Rates up to 428 ppm were observed. Brown et al.^60^ reported that such a MW plasma for NH_3_ synthesis operates under mild conditions and offers a modular and energy-efficient alternative to traditional NH_3_ synthesis methods while allowing for better scalability and integration with renewable energy sources. Nevertheless, the reported production rates are much lower than those reported by many other authors.

### 6.3 DC and Low-Frequency AC Plasma

Glow discharge (GD) plasma is generated by direct current (DC) or alternating current (AC) at low frequencies 50–60 Hz and low pressures (1.3–13 mbar). The gas temperature is moderately low and often close to room temperature, depending on the reactor configuration and power input. A stable glow is sustained between the two electrodes, resulting in visible plasma with a distinct glowing area between the electrodes.

The synthesis of NH_3_ using GD plasma was first reported by Donkin in 1873.^61^ Systematic studies were conducted much later by Brewer et al. in 1929–1930 using a batch process.^62,63^ Eremin et al. (1968–1969)^64,65^ highlighted the potential effect of reactor walls on the NH_3_ synthesis. He pioneered the addition of catalysts to the plasma reactor walls. After 1980, extensive research has discovered the applicability of numerous metal oxides, alloys, and transition metals as catalysts, either in a packed bed reactor or on the reactor walls and electrodes, indicating the feasibility of association catalysts with GD plasmas for the synthesis of NH_3_.^35,36,66–68^

In 1986, Sugiyama et al.^36^ reported the synthesis of NH_3_ using MgO, Cu, CuZn alloys, stainless steel (SS), and acidic oxide catalysts. Synthesis was performed under the following conditions: H_2_/N_2_ ratio of 3:1, pressure of 13 mbar, current of 6 mA, and high DC electric voltage was applied to generate plasma. The authors observed that the MgO catalyst in the GD plasma exhibited catalytic activity, which was not observed in traditional thermal NH_3_ synthesis. However, the authors did not present specific quantitative results.

Venugopalan et al.^66^ reported the synthesis of NH_3_ using different electrode materials for sustaining AC plasma at the following discharge parameters: power 200 W, current 15 mA by applying 12–14 kV, frequency 60 Hz, and gas pressure of 9 mbar. The authors stated that the NH_3_ synthesis was influenced by the electrode materials, which dominated the reaction processes in the plasma. The order of the NH_3_ formation efficiency was Pt > SS > Ag > Fe > Cu > Al > Zn. This order differs from that reported by Wang et al.^24^ Venugopalan et al.^66^ found a correlation between the electron work function (Figure 15) of the electrode material and the NH_3_ yield, demonstrating that higher work functions lead to improved efficiency in producing ionic and radical precursors.

**Figure 15:**
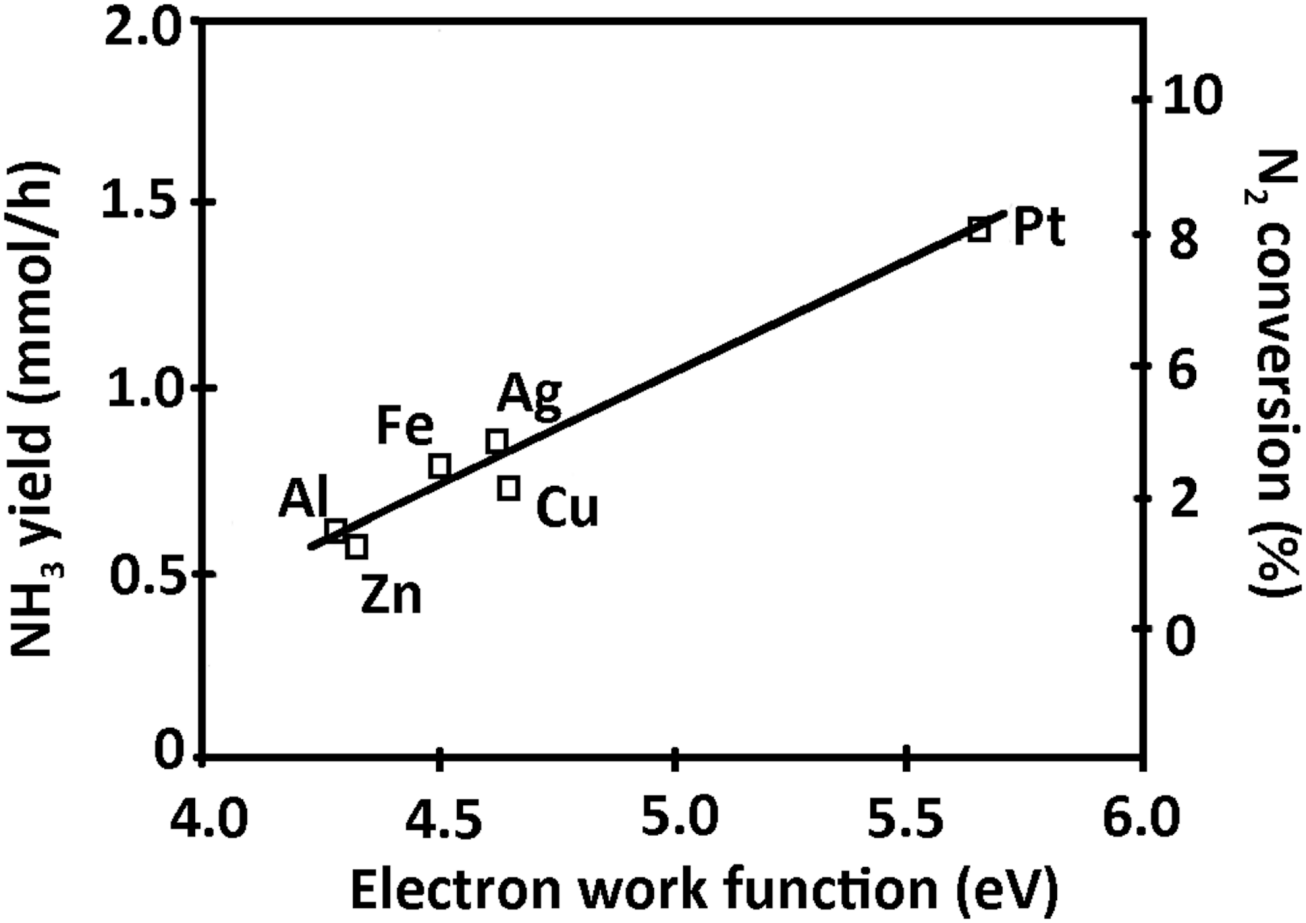
Nitrogen conversion and NH_3_ yield as a function of electron work function of the electrode Material, Reproduced or adapted with permission from ref 66. Copyright 1983, Springer Nature.

According to the author, platinum, which has a higher work function, increased the potential gradient near the cathode, thus enhancing the development of ionic and radical precursors^66^ essential for the synthesis of NH_3_. They reported that NH_3_ is primarily formed at the reactor wall, which acts as a catalytic surface. This result is consistent with the statements in Section 2. The final conclusion of the above-mentioned research groups^34,64^ on the reactor walls is that the formation of NH radicals, as well as reactions of NH radicals with H atoms on the reactor walls, are assumed to be responsible for NH_3_ production from N_2_ and H_2_.

### 6.4 Radio-Frequency (RF) Plasma

RF plasma is generated by a radio-frequency power source, usually operating at 13.56 MHz, and is often sustained at low pressures. It can be capacitively coupled (CCP) or inductively coupled (ICP). RF fields boost an oscillating electric field that sustains the plasma even without direct contact between the electrodes and plasma. In ICP-RF plasmas, an electric current flows through a coil that can be looped over the plasma reactor or placed on top of it. The electric field penetrates deeper into the plasma because the skin effect is less pronounced for RF than for microwaves. Still, the penetration depth is limited, so the bulk plasma is almost free from electrical fields. The main advantage of electrodeless RF plasma is its maintenance without direct electrode contact, which reduces problems such as electrode erosion caused by bombardment with N_2_^+^ ions. Few experimental studies have been performed on NH_3_ synthesis using low-pressure RF discharges. Low-pressure RF plasmas can be created in large volumes, provided that they operate near or below the Paschen minimum. They are widely used in some industries (e.g., in the semiconductor industry) and may also be adapted for industrial NH_3_ synthesis.

Yaale et al.^69^ reported the effects of a tungsten catalyst on NH_3_ production for different N_2_/H_2_ ratios. They used a plasma reactor system shown in Figure 16, composed of a cylindrical quartz tube with an outer diameter of 35 mm, an inner diameter of 31 mm, and a length of about 1.4 m, connected to a 350 mm long waveguide surfatron plasma source, according to the author. Plasma was created with a 13.56 MHz RF generator at a power of 120 W. Equal amounts of H_2_ and N_2_ gases (50% N_2_ and 50% H_2_) were introduced into the quartz tube. The pressure inside the discharge tube was monitored with a baratron. Inside the tube, a 50 cm × 10 cm rolled tungsten foil (purity 99.97%) was used as the catalytic surface. The catalyst could be heated up to a temperature of 1000 °C using a furnace. With this setup, the NH_3_ yield reached 32% (or 320,000 ppm as recalculated) at an N_2_/H_2_ ratio of 25/75%. In the absence of the catalyst, the NH_3_ yield was only 12%. These values are much larger than those reported for DBD plasma. Figure 9b shows the typical NH_3_ yield below 1%.

**Figure 16:**
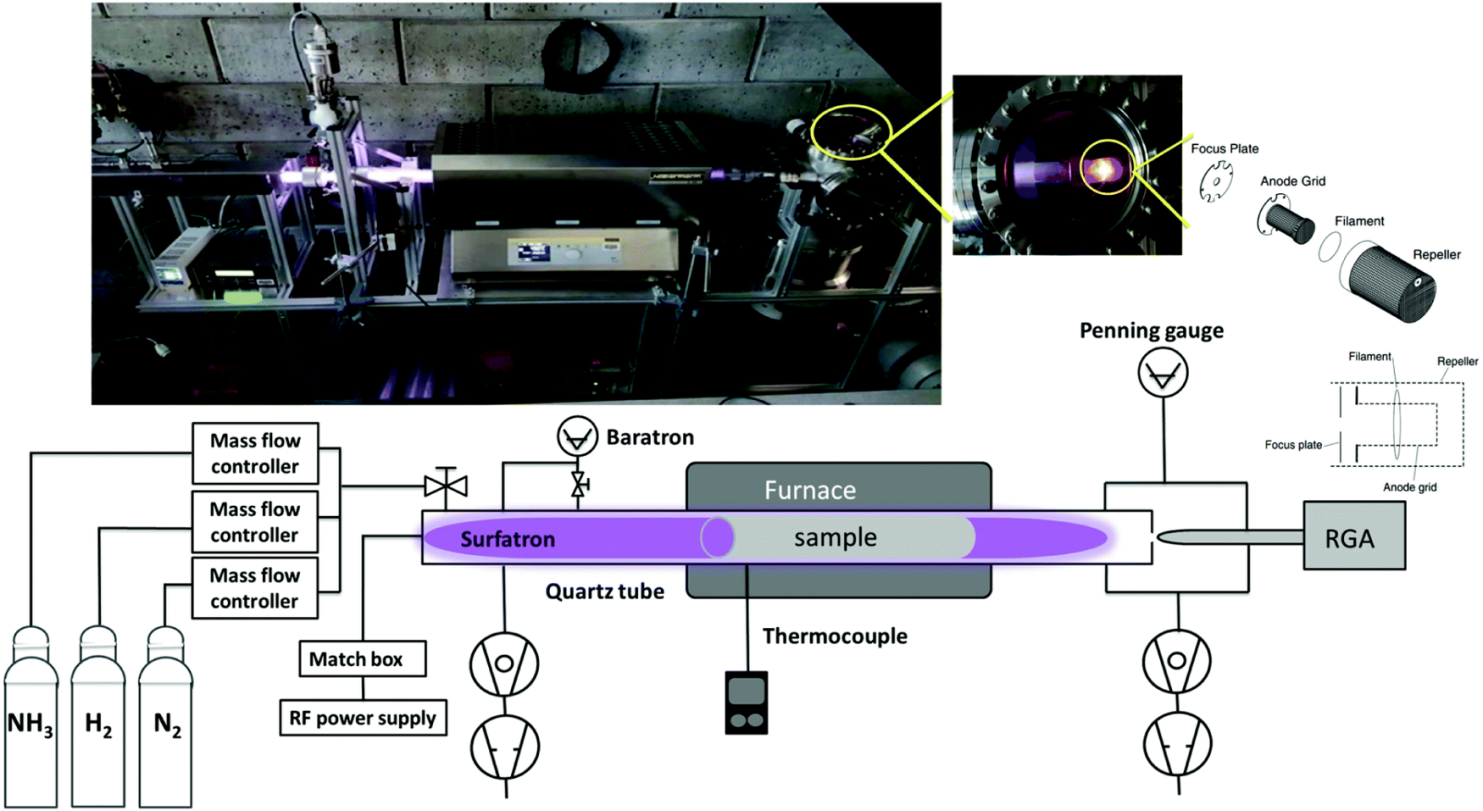
Schematic and real picture (on top) of the RF plasma reactor which enabled conversion rate as larger as 32%, Reproduced from ref 69. Available under a CC-BY 3.0 license. Copyright 2019, M. Ben Yaala.

Shah et al.^70^ studied the NH_3_ synthesis using gold (Au) mesh as a catalyst in inductively coupled (ICP) RF plasma operated at low pressure and experiments were performed in an in-house built plasma reactor (plasma glow zone 80–86 cm). The other parameters were the following: a gas temperature of 400 °C, a pressure of 0.35 mbar, a 1:4 N_2_:H_2_ ratio to the reaction chamber using mass flow controllers, and an RF power of 300 W. The power consumed by the vacuum pump was calculated using a digital clamp-on meter. The power was 169 W, which is approximately 20% (approximately) of the total power consumed during operation (RF Power Supply + Vacuum Pump) at an output plasma power of 300 W. They demonstrated that the NH_3_ synthesis yield in plasma in the presence of a Au catalyst was 19%, and only 3.7% was achieved without a catalyst. The authors also tested other transition metals such as Pd, Ag, Au, Fe, and Cu. The metals were purchased in the form of 0.1 mm wires. Gallium was coated onto inert glass capillaries and loaded into the reactor. The mass of the loaded catalyst was 1 g for all catalysts. Experiments were performed for input powers varying from 50 to 300 W in steps of 50 W. The produced gases were bubbled into deionized water and titrated with dilute sulfuric acid using phenolphthalein as the indicator. The corresponding NH_3_ yields for Pd, Ag, Au, Fe, and Cu were 17.6, 18.6, 19.1, 11.8, and 16.7%, respectively. This order differs from those reported by Wang et al.^24^ or Venugopalan et al.^66^ The yield of 19% surpasses that of the Haber-Bosch process. However, the energy yield of 0.2 g-NH_3_/kWh and the energy cost of 229 MJ/mol still need to be greatly improved to be competitive with H–B. Table 3 compares the conventional HB process with NH_3_ synthesis in RF plasma.

**Table 3:** Comparison of Haber–Bosch (H–B) versus RF Plasma Process for NH_3_ Synthesis

parameters	Haber–Bosch (H–B)	RF plasma^70^	RF plasma^69^
NH_3_ yield	8–15%	19.1%	32%
energy yield (g- NH_3_/kWh)	500	0.19	0.32
energy cost (MJ/mol)	0.48	229	–^a^
set up	small scale not viable	small scale viable	small scale viable
plant size for economy	more than 100 ton/day needed	potential to be adaptable for small scale plants in combination with renewable electricity sources	potential to be adaptable for small scale plants in combination with renewable electricity sources
*temperature*	**450–600 °C**	**25–400 °C**	**25–1000 °C**
pressure	150–350 bar	0.001 bar^b^	0.001 bar^b^
catalyst	iron catalyst	gold catalyst	tungsten surface catalyst
catalyst poisoning	catalyst needs to be regenerated	hydrogen plasma species keep the catalyst clean	hydrogen plasma species keep the catalyst clean

a Insufficient data for recalculation from the original manuscript.

b Recalculated.

Shah et al.^71^ investigated inductively coupled RF plasma for NH_3_ synthesis when employing catalyst Ga, In, and their alloys. Experiments were performed in an in-house built plasma reactor. The gas temperature was about 400 °C, the pressure 0.35 mbar, and the catalyst mass 1 g. The nitrogen and hydrogen flow rates were 4 and 16 sccm, respectively. The plasma was characterized using optical emission spectroscopy (OES), and the catalyst surfaces were characterized by scanning electron microscopy (SEM). A maximum energy yield of 0.31 g NH_3_/kWh and energy cost of 196 MJ/mol was achieved with Ga–In alloy at the power of 50 W. Granular nodes were observed on the surface of the catalysts, indicating the formation of a GaN intermediate. The authors placed the catalyst bed very close to the RF coil excitation zone. They reported that transition-metal-based catalysts such as Ga alloys, i.e., Ga–In, gave the best NH_3_ yield.

Antunes et al.^72^ studied the production of NH_3_ at low pressure using a 13.56 MHz surfatron-RF plasma generator with an associated impedance-matching network and a wide range of experimental conditions (power, pressure, and temperature). The surfatron was attached to a 1400 mm-long Pyrex chamber. The Pyrex tube was connected via a 2 mm wide PEEK pinhole to a high-vacuum (HV) chamber through which the gas-phase species were pumped away. The gas and plasma pressures (on the order of a few pascals during the experiments) were measured using an MKS Baratron sensor mounted downstream of the source. The highest NH_3_ yields were observed at a power of 120 W, a pressure of 3 Pa, and a gas ratio of 50–50 mol % N_2_–H_2_ (the total flow used in the experiments was set to approximately 1 sccm). The authors used tungsten as the catalyst to increase the ammonia production rate. They observed that the NH_3_ yield decreased with increasing power from 120 to 300 W. This may be explained by overheating of the catalyst. These experiments thus show that decreasing NH_3_ or its saturation beyond 120 W is a waste of the input energy, as increasing the RF power does not lead to higher NH_3_ production. Another explanation could be the destruction of the ammonia produced on the surfaces by dissociation with plasma electrons. The surface properties of the tungsten catalyst, such as the adsorption energies and reaction intermediates, significantly influence the reaction mechanism and overall yield. Variations in electron density, electron temperature, and gas composition allow the catalytic properties of the tungsten catalyst to be fine-tuned more effectively than changing the temperature of the gas. The tungsten catalyst proved to be stable with consistent catalytic activity under varying plasma conditions in the range of 40–50 mol % N_2_ at 120 W (Figure 17), making it a valuable material in the field of plasma catalysis. It should be mentioned that tungsten (like many other metals) forms a thin oxide film on its surface, but it is probably reduced by treatment with hydrogen-containing plasma.

**Figure 17:**
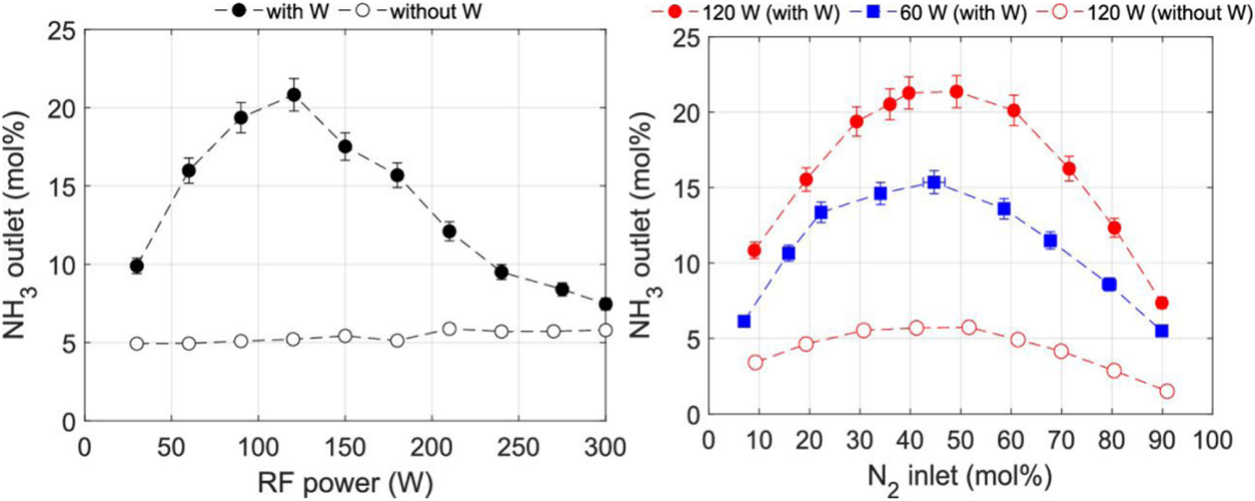
X_NH3_ as a function of the RF power. Empty circles, without tungsten; filled circles, with tungsten (left). X_NH3_ as a function of the inlet N_2_ concentration for 120 W (red circles) and 60 W (blue squares). Empty circles, without tungsten; filled circles, with tungsten (right), Reproduced from ref 72. Copyright 2021, American Chemical Society.

Kim and Mangolini^73^ NH_3_ synthesis using a fine stainless steel or copper mesh as a catalytic surface, with a pulsed CCP-RF plasma sustained at low pressure. The authors found that a proper catalyst, in combination with pulsed plasma, can improve NH_3_ and energy yields. The experimental setup for the plasma reactor used is shown in Figure 19. It consisted of a cylindrical quartz tube (2.54 cm diameter), an RF-driven plasma at a frequency of 13.56 MHz running in a pulsed mode (2, 10, 20, and 30 ms), duty cycle (5, 25, 50, and 75%), and a gas pressure of 0.65 mbar. The total flow rate of the N_2_–H_2_ mixture was kept constant at 20 sccm. Different H_2_:N_2_ ratios were studied in terms of their effects on the NH_3_ yield and efficiency using OES to estimate atomic densities (Figure 18) and a residual gas analyzer (RGA) to quantify NH_3_ yields (Figure 19).

**Figure 18:**
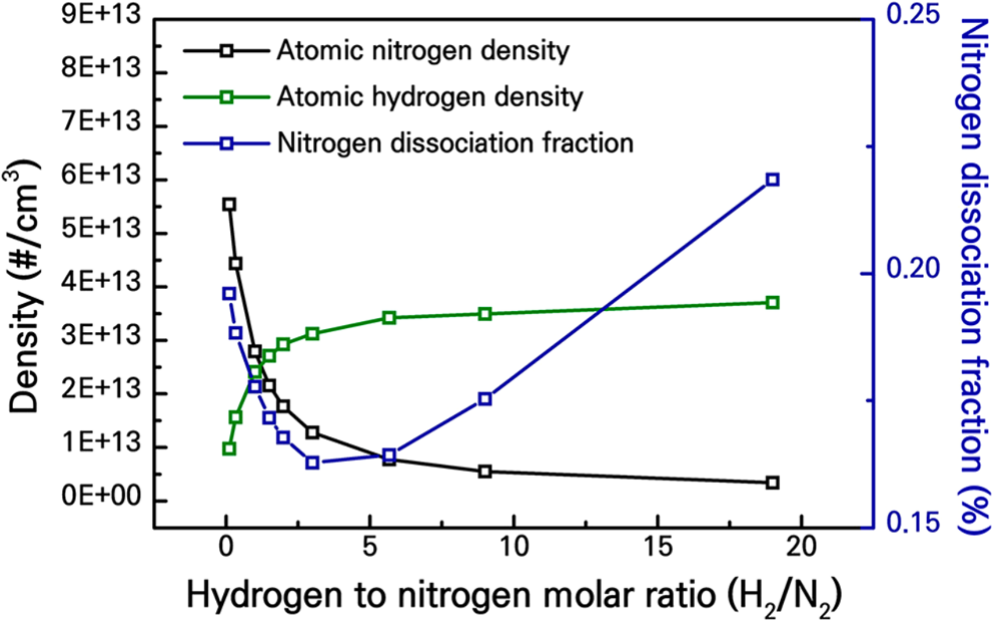
Densities of atomic nitrogen and hydrogen and nitrogen dissociation fraction as obtained through the optical emission spectra and Bolsig+ calculations, Reproduced from ref 73. Copyright 2022, American Chemical Society.

**Figure 19:**
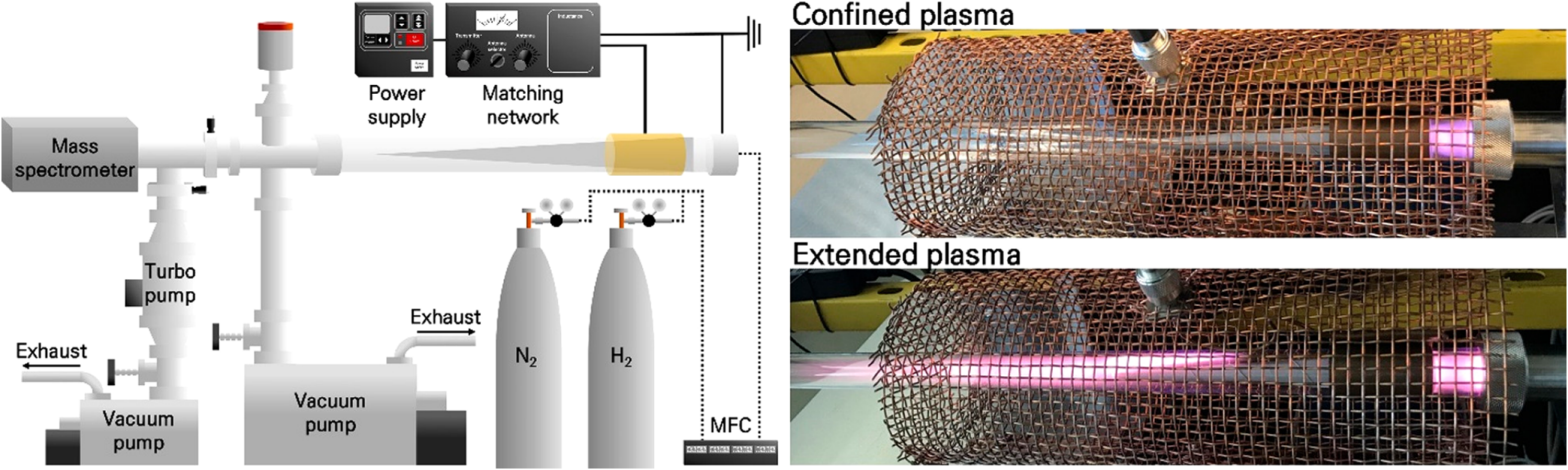
Schematic of the flow-through RF-driven plasma system for the NH_3_ synthesis (left). Photographs of the plasma while in operation (right). The maximum yield of NH_3_ and the corresponding minimum in energy cost is observed when the plasma is confined within the mesh that acts as a catalyst. The NH_3_ yield drops as the plasma extends downstream of the catalyst mesh, Reproduced from ref 73. Copyright 2022, American Chemical Society.

Kim and Mangolini performed Density Functional Theory (DFT) and Born–Oppenheimer molecular dynamics (BOMD) simulations of different radicals (such as NH and NH_2_) on a copper surface catalyst and again found that hydrogen is crucial for the stabilization of any chemisorbed nitrogen. Thus, H_2_ plays a key role in the activation of NH_3_ in the plasma. Without H_2_, the nitrogen atoms recombine rapidly and the nitrogen molecules desorb from the surface. According to the authors, more H_2_ enhances the stability of adsorbed N_2_, enabling a more efficient utilization of plasma-activated nitrogen (atomic and vibrationally excited nitrogen) to form NH_3_. Atomistic simulations suggest that this is related to the role played by hydrogen in stabilizing the adsorption of nitrogen onto the catalyst surface. They compared nitrogen dissociation and vibrational excitation, as it is assumed that both processes lead to NH_3_ formation when interacting with the catalyst surface. According to the author, the efficiency of N_2_ fixation increases up to 8.4% under a greater hydrogen flow (N_2_ flow of 1 sccm and H_2_ flow of 19 sccm). However, although higher ratios improve the yield, they also lead to higher energy costs, which is a drawback that must be managed for optimal NH_3_ synthesis. The large yields could be explained as a consequence of pulsed discharge, resulting in the removal of synthesized ammonia from the discharge volume during the plasma-off time.

In a recent publication by Yang et al.,^74^ the authors reported a nanosecond pulse discharge and a hybrid nanosecond pulse/RF discharge in a plane-to-plane geometry for plasma-catalytic NH_3_ synthesis using Ni/γ-Al_2_O_3_ or Co/γ-Al_2_O_3_ as catalysts. A heated plasma flow reactor was used for the experiment. A 10% H_2_–N_2_ mixture flowed through the reactor cell at a total flow rate of 100 sccm and a pressure of 190 Torr. The benefit of nanosecond pulses is the short duration of discharge, which allows for efficient energy transfer, endorsing the dissociation of molecular gases into reactive species without excessive thermal input. In experiments conducted in a preheated H_2_–N_2_ mixture with and without catalysts, two approaches were compared: a one-step continuous nanosecond pulse discharge (the plasma is sustained by a custom-built nanosecond pulse generator with a peak voltage of up to 12 kV and pulse duration of approximately 100 ns) operated continuously at a pulse repetition rate of 1 kHz. and a two-step process, in which the catalyst was preactivated in pure N_2_ before being exposed to the H_2_–N_2_ mixture. First, preactivation of the catalyst was performed for 30 min in the reactor operating at a 1 kHz ns pulse discharge in pure N_2_ flow (resulting in no detectable NH_3_ yield, as expected). Then, it was exposed to a 10% H_2_–N_2_ flow while the discharge was switched off. This is referred to as the two-step process, that is, the plasma-off process. The Co and Ni catalysts enhanced the formation of NH_3_ because of their catalytic effect and ability to enable surface reactions among the adsorbed N and H atoms. The nanosecond pulse discharge efficiently activated the catalyst surfaces and enhanced adsorption and reaction, ultimately leading to higher NH_3_ yields. Moreover, the RF discharge was shown to improve the NH_3_ yield by approximately 20% as compared to the ns pulse discharge operating alone, measured both in the empty reactor and over the Ni catalyst. The authors concluded that both the vibrational excitation of N_2_ and surface reactions are crucial for increasing the productivity of NH_3_ formation.

In a recent publication, Vervloedt et al.^75^ reported that nitrogen and hydrogen contributed to the formation of NH_3_ when they were mixed with helium RF plasma. Plasma was characterized by Fourier transform infrared absorption spectroscopy (FTIR), OES and kinetic modeling. The plasma volume (26 mm × 13 mm × 1 mm) was located in the central section of a rectangular gas channel (54 mm × 14 mm × 1 mm). In the plasma reactor, 250 sccm of helium was mixed with 2.5 sccm of nitrogen and hydrogen. The pressure was 1 atm. The power was 4 W and the applied voltage was 350 V. The reactor was enclosed in a low-pressure vessel to minimize air contamination. The authors used Fe, Pt, and Cu catalysts supported on sandblasted glass. These catalysts were tested for NH_3_ formation at surface temperatures up to 150 °C. The authors found that the introduction of the catalyst into the plasma reactor significantly improved NH_3_ formation and doubled the yield (compared with the reactor without a catalyst). The authors also developed a kinetic model for NH_3_ synthesis, which shows that small changes in the reduced electric field can explain the changes in NH_3_ formation rates between catalytic and noncatalytic surfaces. The future focus of the authors is on the precise characterization of the interface between the plasma and catalytic surfaces, together with *in situ* measurements of surface coverage, to increase the understanding of surface kinetics. The reported yield was as low as 32 ppm, which is orders of magnitude lower than the values reported by other authors using RF discharges.

Shah et al. and Carreon et al.^76^ tested a Ni-based metal–organic framework (Ni-MOF-74, 0.2 g) for NH_3_ synthesis using an in-house built RF plasma reactor. For the catalyst system, Ni-MOF-74 showed an NH_3_ yield of 52,500 ppm under the following reaction conditions: H_2_:N_2_ ratio of 4:1 (4 and 16 sccm) and power of 200 W. The vacuum pressure in the chamber was 0.3 Torr with N_2_ and H_2_ flow. These MOFs had a large surface area and porous structure, which resulted in efficient mass transfer of reactants and products for higher NH_3_ formation. In addition, MOFs with metallic sites acted as active catalytic centers that endorsed the adsorption and reaction of hydrogen and nitrogen. Moreover, MOFs can also reduce hydrogen recombination at the surface, thus increasing the accessibility of reactive hydrogen, which ultimately increases NH_3_ yield.

Low-pressure RF and MW plasma reactors enable the generation of uniform plasma in a large volume, as explained in Section 2. This is a significant advantage compared to DBD reactors, which usually produce filamentary plasma. Table 4 summarizes the NH_3_ synthesis yields for RF, MW, and glow-discharge plasmas. The reported energy and NH_3_ yields for the RF plasma are shown in Figure 20, and are similar to those for DBD in Figure 9. In addition, the points are scattered; therefore, no direct conclusion can be drawn. Unfortunately, for MW plasma, there is insufficient data in the literature to make an independent comparison. Still it is interesting that the values in Figure 20b are much larger than in Figure 9a. At a glance, one would conclude that RF discharges provide higher ammonia yields, but one should also consider the fact that all DBD plasmas reviewed in this article were sustained at atmospheric pressure, while RF and MW nonequilibrium plasmas, are usually sustained at low pressures.

**Figure 20:**
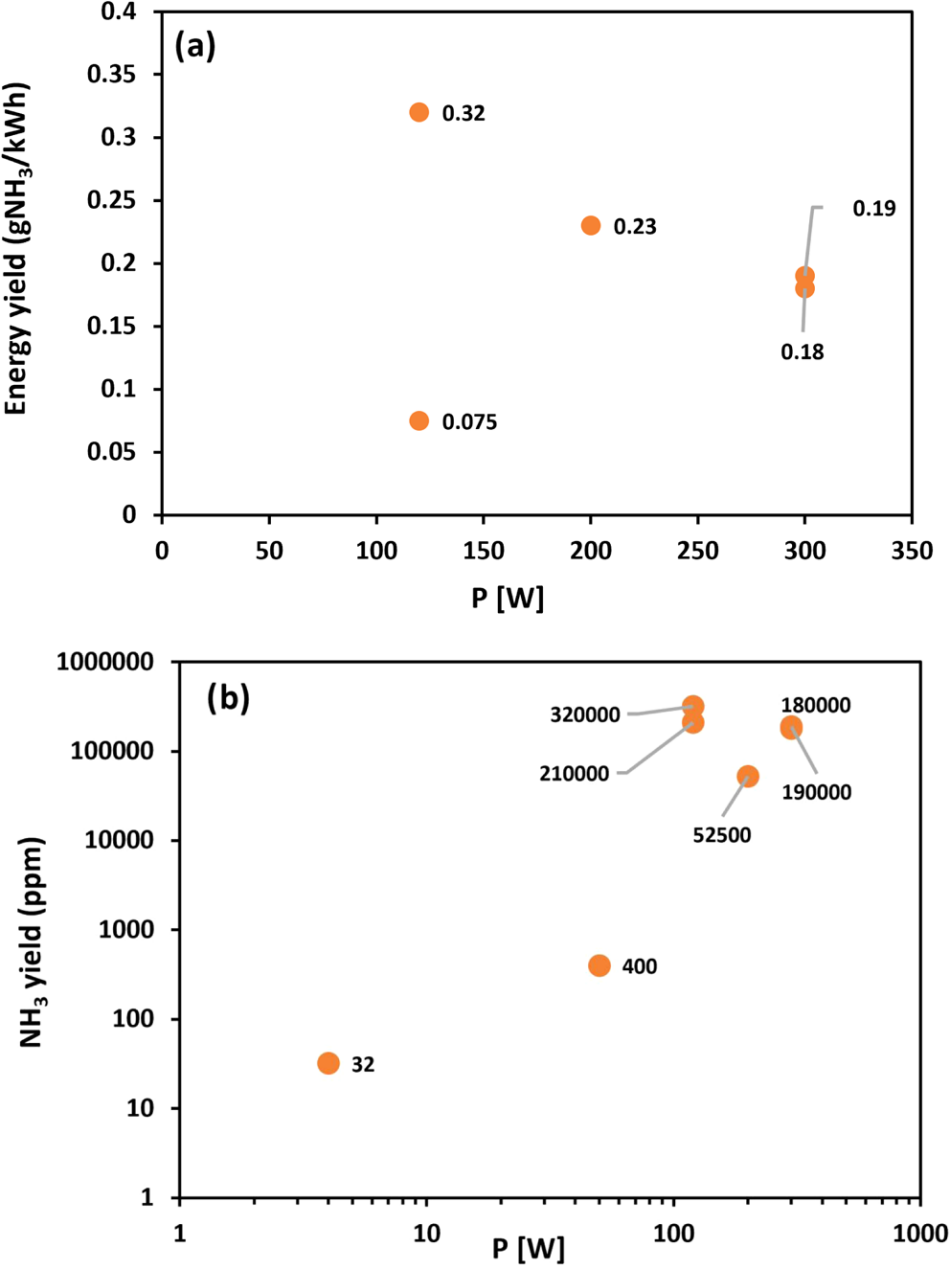
Energy yield (a) and NH_3_ yield (b) versus power for RF plasmas.

**Table 4:** Comparison Table for NH_3_ Synthesis by RF, MW, and Glow-Discharge Plasma^a^

s. no	catalyst	plasma reactor type	H_2:_N_2_	*Q*_gas_ (sccm)	*p* (Pa)	*f* (kHz)	*P*_d_ (W)	NH_3_ yield (ppm)	note	energy yield (gNH_3_/kWh)	refs
1	no catalyst	MW plasma			10^5^		1100	31,000^d^ (1823.5 μmol/g)		0.04	^58^
2	Co/Al_2_O_3_	MW plasma			10^5^		NA	1100^d^ (64.7 μmol/g)	catalyst was heated to 600 °C	0.01	^59^
3	Fe wires	MW plasma			650		130	1.5 μmol g^–1^		NA	^37^
4	stainless steel wall	MW plasma	1:3		600		200	NA		NA	^41^
5	Fe-based catalysts	MW plasma	1:1			2.54 GHz	300	420.9 μmol min^–1^g^–1^	temperature of 450 °C and extra Ar gas	0.04^d^	^60^
6	MgO catalyst	Glow-discharge plasma	3:1		1300		6 mA	NA			^36^
7	Pt electrodes	Glow-discharge plasma	5:2		900	60 Hz	200	1.46 μmol h^–1^		0.13^d^	^66^
8	tungsten surface	in-house built RF plasma reactor	3:1		2		120	320,000^d^ (18,823.5 μmol/g)	temperature of 1000 °C	0.32	^69^
9	gold mesh	low pressure, RF plasmas	4:1	20	30		300^c^ (400 °C)	190,000^d^ (11,176.4 μmol/g)	temperature of 400 °C	0.19	^70^
10	Ga–In cat	low pressure, RF plasmas	4:1	20	30		300^c^ (400 °C)	180,000^d^ (10,588.3 μmol/g)	temperature of 400 °C	0.18	^71^
11	tungsten	low pressure, RF plasmas	1:1		3		30	210,000^d^ (12,352.9 μmol/g)		0.075	^72^
12	cone-shaped metal mesh	RF-pulsed plasma.	0.1–19 (molar ratio)	20	66		150	NA		7.7	^73^
13	Ni or Co catalyst on alumina ceramic powder	ns pulse/RF plasma	10% N_2_–H_2_ and 10% H_2_–Ar^b^	100	25,270	1	50	400 (23.5 μmol/g)	temperature of 300 °C and extra Ar gas	0.001^d^	^74^
14	Fe nanoparticles	atmospheric RF discharge plasma	250 sccm of He+ 2.5 sccm of N_2_/H_2_ ^b^		10^5^		4	32 (1.88 μmol/g)	extra He gas	0.1	^75^
15	Ni-MOF-74 (0.2 g)	RF plasma^c^	4:1	20	40		200	52,500^d^ (3088.3 μmol/g)		0.23	^76^

a NA-not available data.

b Note.

c Extra heating.

d Recalculated.

Figure 21 shows the energy and NH_3_ yields versus the reported pressure. Many authors have not provided complete details, resulting in a limited number of data points in the diagrams shown in Figure 20. We selected logarithmic scales in Figure 20 because of the enormously large variations in both pressure and yield.

**Figure 21:**
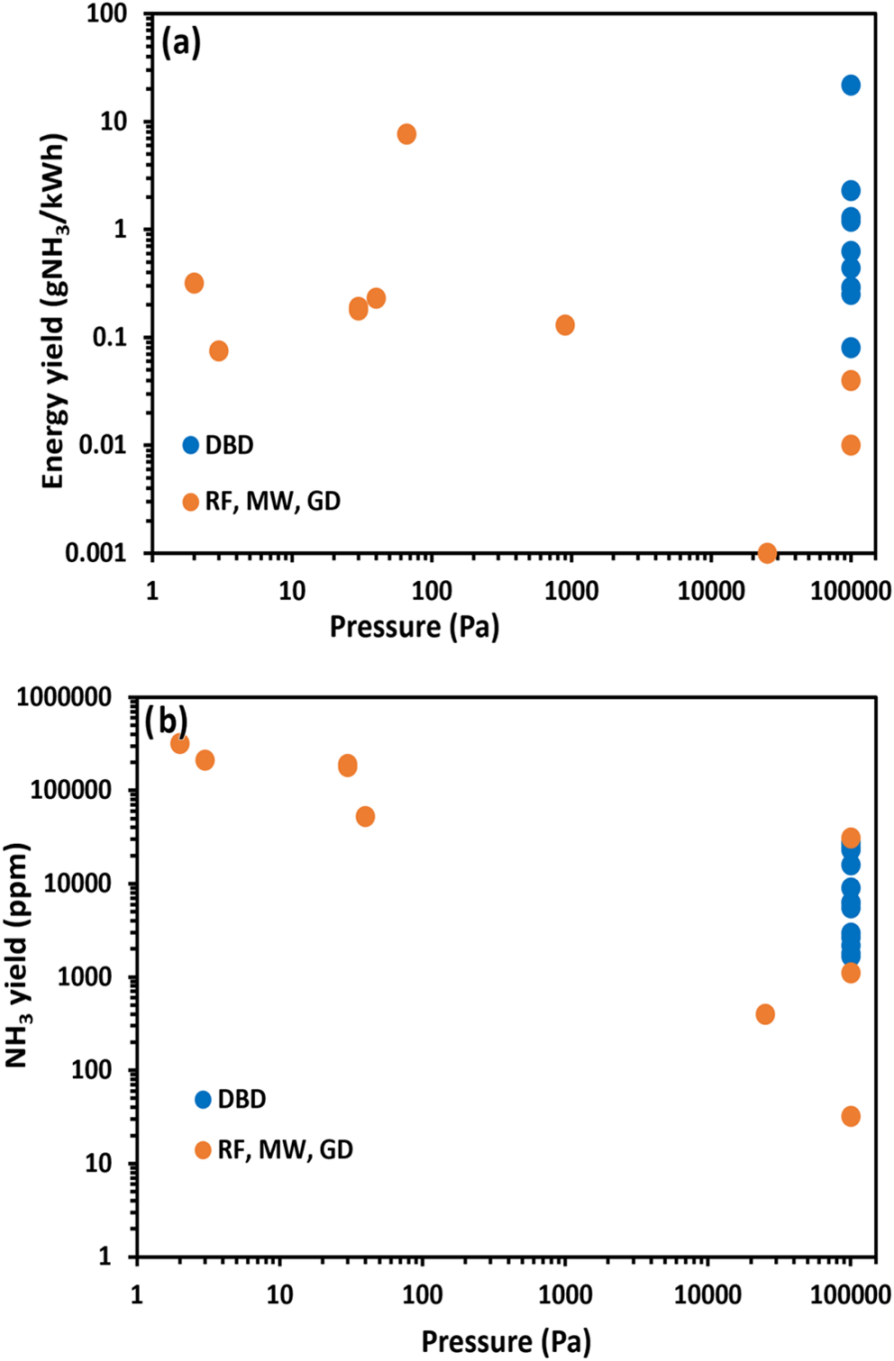
Energy yield (a) and the ammonia yield (b) versus the pressure in the plasma reactor.

We first discuss the energy yield, which is shown in Figure 21a. Except for one point at a pressure of 66 Pa, all reported data for the low-pressure range are approximately 0.1 g/kWh, one can conclude that the yield in the low-pressure range does not depend much on the pressure. As mentioned in Section 2, low-pressure plasmas are governed by the loss of plasma radicals on surfaces (and not in the gas phase). A detailed explanation of this effect is provided in the classical literature on the advantages of low-pressure plasmas.^77^ The pressure at which the loss of radicals by surface and gas-phase reactions equalizes is a few 10 mbar, i.e., a few 1000 Pa. All points in Figure 21a are well below this boundary, so it is not surprising that the plasma-catalytic conversion yields do not depend significantly on the pressure in the range of pressures used by the reviewed authors, that is, between 1 and 1000 Pa. The yields in the low-pressure range are still far from the yield of NH_3_ in the H–B process, which is of the order of 10^2^ g/kWh (see Figure 23).

There are three points in the high-pressure range for RF, MW, and glow discharge plasmas and more for DBD plasmas in Figure 20a, indicating an energy yield between 10^–3^ and a few 10 g/kWh. In high-pressure ammonia production, there are two main problems that have already been introduced in Section 2: first, the energy spent on sustaining gaseous plasma at atmospheric pressure is predominantly used for heating the gas because of the abundance of three-body collisions, and second, the destruction of ammonia molecules in the gaseous plasma. The channels responsible for this unwanted effect were recently presented by Fedirchyk et al.^78^ Briefly, the NH_3_ molecules in cold gaseous plasma can be dissociated by electron impact and the quenching of nitrogen metastables. In both cases, hydrogen atoms are released, and they are likely to recombine with another H atom and form an H_2_ molecule during three-body collisions with either nitrogen or hydrogen molecules, which abound at atmospheric pressure.

Figure 21b shows the ammonia yield versus the pressure. As shown in Figure 21a, the yield does not depend on the pressure in the low-pressure range, i.e., between 1 and 100 Pa. The pressure independence is again explained by the fact that the loss of plasma radicals in the low-pressure range is predominantly on surfaces; therefore, the frequency of three-body collisions does not influence the ammonia yield. The yield is a few tens of percent, which is comparable to the single-pass yield of H–B. The yield in low-pressure reactors is thus reasonable, but the main problem is the very low density of molecules at low pressure compared to atmospheric pressure. At atmospheric and subatmospheric pressures (right-hand points in Figure 21b), the yield is lower, that is, between 0.003 and 3%. Because the role of the gas residence time correlates more with the pressure and flow rate, the authors did not specify the residence time and did not provide the information required for their calculation, namely, the gas flow rate used in their experiments and the dimensions of the plasma region.

In Figure 22, there is a correlation between the energy and NH_3_ production yields of the different catalysts reported by all the authors reviewed in this paper. The diagram is on a log–log scale and shows huge differences between the results reported by different authors, but there is no statistically significant correlation.

**Figure 22:**
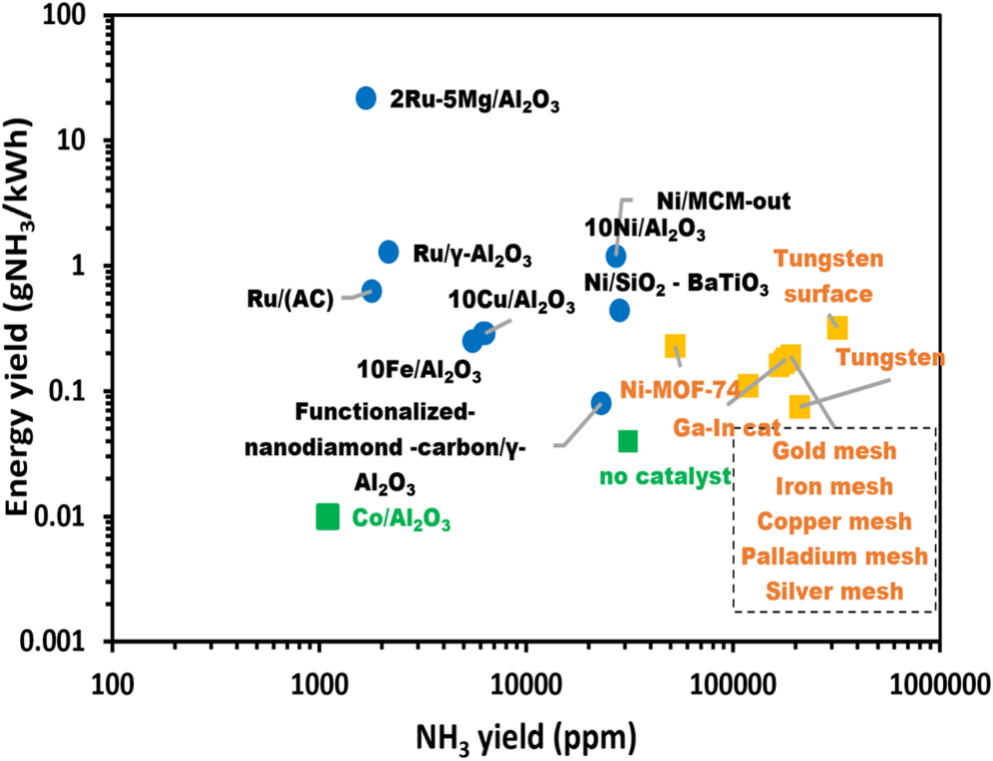
Energy yield vs NH_3_ yield (DBD- blue, MW-green dot and RF plasmas-yellow) of different catalysts.

## 7 Ammonia Decomposition

In the presence of plasma, the ammonia formed can undergo decomposition, which affects NH_3_ yield and efficiency. This has also been observed in the H–B process, where harsh conditions, including high temperature (t) and high pressure (p), can lead to decomposition of the NH_3_ produced. Several research groups^40,79–81^ have investigated how the decomposition of ammonia can be avoided, using packed catalysts in plasma-based reactors.

Rouwenhorst and Lefferts research group^81^ investigated the *in situ* adsorption of NH_3_ with zeolite 4A during plasma-chemical NH_3_ synthesis increases the NH_3_ yield compared to stationary plasma-assisted NH_3_ synthesis. This can be attributed to the adsorption of NH_3_ on the zeolites, which suppresses the decomposition of NH_3_ in the plasma phase. This improves the energy yield during NH_3_ synthesis by a factor of 2. Maria L. Carreon’s research group^40^ investigated the alkaline earth metal perovskites MTiO_3_ (with M = Mg, Ca, Sr and Ba) using for the ammonia synthesis and decomposition rates. Among these, MgTiO_3_ showed a very good NH_3_ synthesis rate of 12.24 μmol min^–1^ m^–2^ at 20 W plasma power. The authors also discovered the NH_3_ decomposition reaction due to the possibility of the importance of the reversible reaction due to electron collision with the formed ammonia molecules. Interestingly, they found that ammonia decomposition increased with increasing plasma power. This indicates the advantage of low plasma power operation and the need to develop plasma reactors where the newly formed ammonia molecules can be removed from the reaction system to avoid further electron collisions.

The Aihara research group^80^ found that a Cu-based catalyst is more efficient in preventing ammonia decomposition than an iron-based catalyst, resulting in a higher ammonia formation rate on the Cu catalyst. Another researcher, Wang et al.,^79^ has provided direct evidence that the recombinative desorption of adsorbed nitrogen atoms is the rate-limiting step in the plasma catalytic decomposition of ammonia on metal catalysts with stronger metal–nitrogen bonds (where M stands for metals, such as Ni–N, Cu–N, Fe–N, etc.). Finally, ammonia decomposition may also be associated with packed catalysts, which is also an important aspect. Several processes are crucial for NH_3_ decomposition in plasma, but little is known about the underlying reactions that drive NH_3_ decomposition. Therefore, there is a clear need for more detailed understanding.

## 8 Techno Economic Analysis and Environmental Impact Analysis

The feasibility plasma-assisted catalytic ammonia (NH_3_) synthesis as an industrial process depends not only on its scientific feasibility but also on its economic competitiveness and environmental sustainability. Despite the progress made, the H–B process continues to dominate global NH_3_ production due to its proven efficiency on a large scale and established infrastructure.

In contrast, plasma catalytic NH_3_ synthesis still has a low technology readiness level (TRL). The current energy efficiency of plasma-based processes is well below the H–B benchmark (∼100 g NH_3_/kWh). Recent studies, such as by Rouwenhorst et al.,^82^ reported the state-of-the-art data for a plasma-catalytic NH_3_ synthesis process vs energy consumption (see Figure 23), taking into account gas recycling and NH_3_ separation. These analyses are often based on assumptions for low-pressure and low-conversion systems with solid sorbents - methods that are still being optimized.

**Figure 23:**
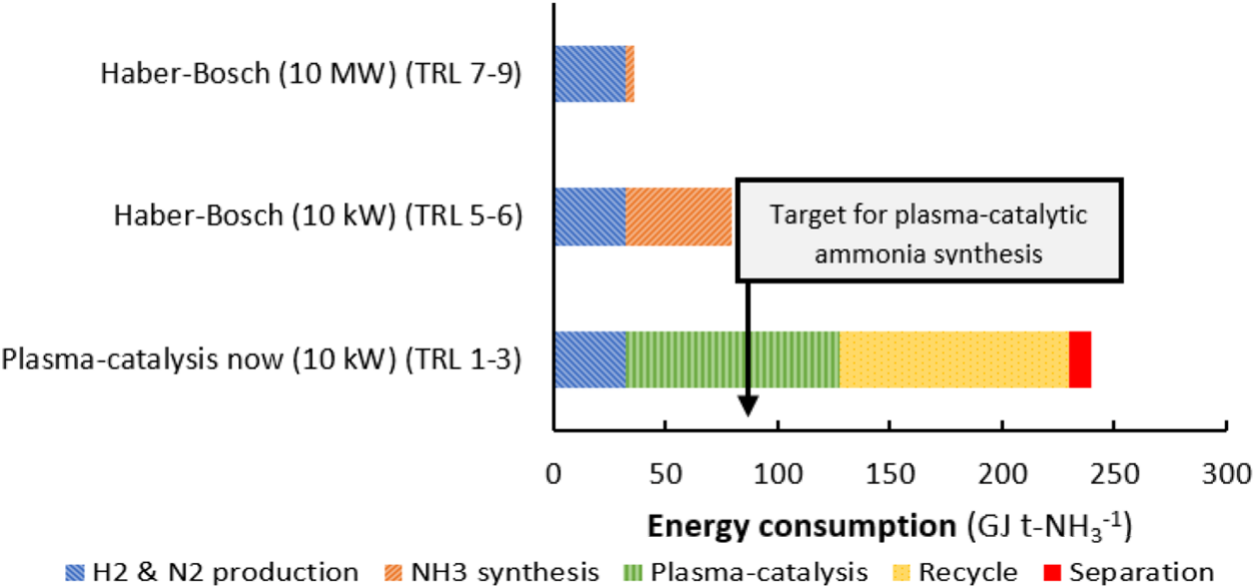
State-of-the-art system for Haber-Bosch and plasma-catalytic ammonia synthesis process versus energy consumption, Reproduced from ref 82.^81^ Available under a CC-BY 4.0 license. Copyright 2020, Kevin H. R. Rouwenhorst.

The separation efficiency remains a major limitation. Conventional condensation techniques are ineffective at low operating pressures due to the vapor pressure of NH_3_ (∼7 bar). Therefore, alternative separation methods, including the use of solid sorbents with strong NH affinity, are essential. Although NH_3_ separation based on sorbents has been known since 1924 (e.g., the formation of ammonium chloride), it requires rigorous techno-economic validation for modern systems.

To become economically viable, plasma-assisted NH_3_ synthesis must be either: Identify niche applications (e.g., remote, off-grid or small-scale NH_3_ production powered by renewable energy), where its flexibility and decentralization offer a competitive advantage. Improved process efficiency through better integration of plasma catalysts and optimization of energy use.

Generally, techno-economic analysis (TEA) comparisons between the plasma-catalytic route and conventional H–B processes are discussed for approximate valuations. For the conventional H–B, CAPEX stands at €159/ton and OPEX stands at €377/ton, but in the case of the CAPEX for plasma ammonia production at €5795/ton, while the OPEX reached a staggering €11,311/ton for small-scale plasma routes.^83^ At present, plasma-catalytic ammonia synthesis remains significantly less energy-efficient and more expensive than both conventional and green HB processes-by approximately 10–30 × in terms of per-ton costs. Current studies suggest that energy efficiency of plasma-assisted NH_3_ synthesis could be enhanced by a factor of 6.^84^ In the future, low-cost renewable energy sources, plasma reactor cost drops, and improved energy efficiency will defeat conventional H–B processes. Finally, this plasma-catalytic route will reach net-zero carbon emissions by 2050.

Given the higher investment requirements and lower energy yield, researchers are considering combining plasma with other renewable energy-based technologies, such as photocatalysis and electrocatalysis, which offer many opportunities to develop innovative synergetic technologies, even though this topic is only just beginning to be explored.^2^

Plasma-assisted NH_3_ synthesis offers potential environmental benefits, especially when powered by renewable energy sources. The process inherently emits no CO_2_, avoids H_2_ derived from fossil feedstocks, and allows for modular decentralized use. These characteristics are in line with the global goals of decarbonization and net-zero chemical production. As the cost of electricity from renewables continues to fall, the case for the sustainability of plasma technology is becoming ever stronger, even though it still lags behind in terms of energy efficiency.

## 9 Challenges, Gaps and Perspectives

Despite its promising potential, plasma catalytic NH_3_ synthesis faces several technical, scientific and practical challenges that need to be overcome for successful scale-up and commercialization.

### 9.1 Scientific and Mechanistic Gaps

However, the reaction mechanisms remain poorly understood. Although it has been hypothesized that vibrationally excited N_2_ species reduce the energy barriers at the catalyst surface, the detailed pathways for NH_3_ formation are not fully understood. There is a lack of quantitative data on the fluxes of plasma species (ions, atoms, and excited molecules) interacting with the catalyst surfaces. Only Zaplotnik et al.^15^ have reported interaction efficiencies (e.g., ∼50% for Ni), while most studies omit such critical parameters. Radiation effects, especially from UV and VUV emissions^85^ in H_2_ and N_2_ plasmas, are still poorly understood but can significantly influence surface chemistry.

The plasma-catalyzed ammonia route leads to a nonequilibrium environment and a highly reactive nature that significantly affects the stability of the catalyst. Intense ion bombardment, UV radiation, reactive radicals, and rapid thermal fluctuations in plasma systems can lead to various degradation mechanisms that affect the long-term performance of the catalyst. The key degradation processes include sintering and poisoning, which need to be understood and mitigated for practical applications in future studies.

### 9.2 Technological and Diagnostic Challenges

Currently, the research community is focusing on NH_3_ synthesis on an industrial scale,^82,84,86–88^ for which we need intensive studies, technologically and engineering aspects.

- In-situ plasma characterization and surface diagnostics are underutilized. Without real-time data on material fluxes and surface reactions, the optimization of reactor designs and catalyst materials becomes largely empirical.
- The surface recombination of N and H atoms to form N_2_ and H_2_ competes with NH_3_ formation and reduces selectivity. Few studies have addressed this competing mechanism or how it can be effectively suppressed.
- Plasma can also cause secondary decomposition of formed NH_3_, especially in high-energy systems such as ICP,^7^ which complicates product stability.

### 9.3 Scale-Up and Reactor Design

The transition from laboratory-scale systems to industrially relevant systems is associated with financial and technical challenges. The main problems include the following.

- Maintaining plasma uniformity.
- Minimizing the energy input.
- Integration of ammonia separation systems under low- pressure conditions.

### 9.4 Future Prospects

To make progress in this area, a multidisciplinary and cross-scale approach is required, which combines the following.

- Advanced plasma reactor technology.
- Rational catalyst design (supported by DFT and kinetic modeling).
- *In situ* plasma and surface diagnostics.
- Integrated techno-economic analysis (TEA) for various application scenarios.

Furthermore, collaboration between academia and industry, supported by governmental and international funding, is crucial for increasing the TRL of this promising technology.

In the long term, plasma catalytic NH_3_ synthesis could revolutionize ammonia production by offering electrified, decentralized, and flexible systems that are well-suited for integration with renewable energy sources. Combined with innovative catalyst development and improved plasma-catalyst synergy, this technology could become a cornerstone of the global transition to carbon-neutral chemical production.

## 10 Conclusions

This review focuses on the recent advances in plasma catalytic ammonia synthesis using different catalyst systems and different plasma sources (i.e., DBD, MW, GA, and RF), as this technology is of great importance for the development of a sustainable hydrogen economy. According to the current state of research, hydrogen transport via ammonia is one of the key routes attracting growing interest in the industry. A representation of the mechanisms of plasma-catalytic ammonia synthesis, reaction conditions, NH_3_ yields, and energy yields are presented. Most studies dealt with Ru-based catalysts for ammonia synthesis using DBD plasma, but also with other metals such as Ni, Cu, Fe, and Pt, with Ru- and Ni-based catalysts showing high ammonia yields. One of the important plasma sources is RF plasmas. The catalysts investigated for RF plasma ammonia synthesis were the tungsten surface, gold mesh, Ga–In, Fe nanoparticles, Ni and Co catalysts, and Ni-MOF-74. After a critical review of plasma catalytic ammonia synthesis using different plasma sources, it was found that the highest NH_3_ yields were obtained using low-pressure RF plasma. A feasible explanation is that low-pressure reactors benefit from predominant surface reactions, whereas atmospheric pressures favor gas-phase reactions, which lead to the loss of plasma species in the gas phase. Another advantage is the easier scalability compared to other plasma sources; therefore, low-pressure RF plasma technology can fulfill the industry’s requirements in the near future. Plasma-based technology driven by renewable energy sources will be suitable for sustainable NH_3_ synthesis and will provide a greener and cleaner alternative to conventional NH_3_ synthesis, leading the ammonia production industry toward sustainability (Figure 1c).

## Data Availability

Data is contained within the article.
